# Psychiatric comorbidities of attention deficit/hyperactivity disorder in Japan: a nationwide population-based study

**DOI:** 10.3389/fpsyt.2024.1359872

**Published:** 2024-10-24

**Authors:** Takashi Okada, Takuma Sotodate, Mari Ogasawara-Shimizu, Nobuhiro Nishigaki

**Affiliations:** ^1^ Department of Developmental Disorders, National Institute of Mental Health, National Center of Neurology and Psychiatry, Tokyo, Japan; ^2^ Department of Psychiatry, Nara Medical University, Kashihara, Japan; ^3^ Japan Medical Office, Takeda Pharmaceutical Company Limited, Tokyo, Japan

**Keywords:** ADHD, autism spectrum disorder, claim database, complications, depression, incidence, prevalence, risk ratio

## Abstract

**Introduction:**

This study aimed to estimate prevalence and incidence of attention deficit/hyperactivity disorder (ADHD) and comorbid relationships between ADHD and other psychiatric disorders in Japan.

**Methods:**

Using the real-world JMDC Claims Database, we conducted a cross-sectional study (analysis years 2017–2021) and retrospective cohort study (observation 2 years before/after the initial ADHD diagnosis; data collection 2005–2021; enrollment 2017–2019). Patients were male or female, aged 0–57 years. Cross-sectional study patients had an ADHD or other psychiatric disorder diagnosis (with or without medication) and were continuously registered in each analysis year; retrospective study patients had an ADHD diagnosis and ≥2 years’ observation before and after diagnosis. Endpoints were annual prevalence and incidence of ADHD in Japan, prevalence and risk ratio of each psychiatric comorbidity in patients with ADHD, prevalence and risk ratios of ADHD in patients with each psychiatric comorbidity, and prevalence of psychiatric disorders before/after the initial ADHD diagnosis.

**Results:**

ADHD prevalence in children/adolescents and adults increased each year from 2017 to 2021. Prevalence in boys was 3.5–4.1 times higher than in girls. Prevalence in adults was lower than in children/adolescents, with a small sex difference. ADHD was highly comorbid with various psychiatric disorders. In 2019, the most common comorbidity in children/adolescents with ADHD was autism spectrum disorder (ASD; 54.4%); in adults, it was mood disorders (60.9%). ADHD prevalence in patients with various psychiatric disorders was higher than in the control population. ADHD prevalence was highest in patients with oppositional defiance disorder among both children/adolescents and adults (77.2% and 69.2%, respectively). In the retrospective cohort study (N = 14,940), the most common psychiatric disorders diagnosed prior to ADHD diagnosis were ASD in children/adolescents (33.9% of patients), and mood disorders and sleep disorders in adults (36.9% and 23.8% of patients, respectively).

**Discussion:**

ADHD was comorbid with various psychiatric disorders in Japan. In children and adolescents with ADHD, ASD was often diagnosed prior to ADHD. Psychiatric disorders, especially mood disorders and sleep disorders, were frequently diagnosed prior to the initial ADHD diagnosis in adults. The likelihood of comorbid ADHD should be considered when diagnosing adult patients with psychiatric disorders.

## 1 Introduction

Attention deficit/hyperactivity disorder (ADHD) is a neurodevelopmental disorder characterized by impaired levels of inattention and hyperactivity/impulsivity that negatively impacts social, academic, and occupational functioning (1–3). ADHD is often first diagnosed in childhood, and some patients have remission or partial remission as they grow; however, others continue to have symptoms at levels that meet diagnostic criteria into adulthood (1–3). In such cases, symptoms can change over time; generally, inattention persists and hyperactive-impulsive symptoms wane (4). While twin studies demonstrated that ADHD is highly heritable with heritability estimates in the range of 70–80% (5, 6), in most cases ADHD is considered to be caused by the accumulation of various genetic as well as environmental risk factors. Consistent with the multifactorial etiology of ADHD, patients with ADHD exhibit considerable variation in profiles of symptoms, type and severity of impairments, and complicating factors (2, 3).

The worldwide prevalence of ADHD in childhood and adolescence has been estimated by meta-analysis to be 7.2% (7) and is higher in boys than in girls (8). Meta-analyses have estimated the prevalence of ADHD in adulthood, based on Diagnostic and Statistical Manual of Mental Disorders, Fourth Edition criteria, to be 2.5–2.8% (9, 10). Additionally, the prevalence of ADHD has been shown to vary according to geographic location (11) and national income level (9). However, the prevalence of ADHD in Japan has not been fully investigated, and evidence for nationwide estimates is limited to date. Epidemiological surveys conducted at the municipal level, which provided a more accurate estimate of the prevalence of ADHD, have reported that the ADHD prevalence in children in Japan is 5.8% (12). Nationwide studies using hospital administrative (13) and claims-based databases (14) showed a gradual increase in diagnosis of ADHD in children and adolescents in Japan from 2012 to 2018 and from 2005 to 2015, respectively. In an epidemiological study in Hamamatsu city in central Japan, the prevalence of ADHD in adults was estimated to be 1.65% (15). There have been no nationwide studies on the prevalence of ADHD in adults in Japan.

People with ADHD often have various psychiatric comorbidities. Previous studies have shown that ADHD often coexists with various psychiatric disorders in childhood, including mood disorders, anxiety disorders, conduct disorders, oppositional defiant disorder (ODD), learning disorders, Tourette syndrome, borderline personality disorder, and autism spectrum disorder (ASD) (16, 17). Twelve-month comorbidities in adults with ADHD were examined using the results of the United States National Comorbidity Survey Replication (18) and the World Health Organization survey (9); these analyses concluded that adults and adolescents with ADHD had a high risk of psychiatric disorders such as anxiety disorders, mood disorders, ODD, and substance use disorders. The coexistence of psychiatric comorbidities imposes an additional burden on patients with ADHD. ADHD itself is associated with an increased risk of suicide attempts (19), and when combined with comorbidities, this risk escalates further. In addition, ADHD is associated with an increased risk of all-cause and premature mortality (20), and psychiatric comorbidities contribute to this increased risk. The prevalence of psychiatric disorders including ADHD has been shown to vary by geographic location, national income level, and cultural differences (21). Comorbidities of ADHD may also differ depending on the temperament and culture of the people. Therefore, it is important to determine the prevalence and comorbidities of ADHD in individual countries or cultures. However, psychiatric comorbidities associated with ADHD have not been widely investigated in Japan. Therefore, we conducted a nationwide claims-based study to identify the prevalence and incidence of ADHD and associated psychiatric comorbidities in Japan using a cross-sectional study design. In addition, using a longitudinal study design, we examined whether ADHD or other psychiatric disorders are diagnosed first.

## 2 Materials and methods

### 2.1 Study design

This was a cross-sectional study and a retrospective cohort study for longitudinal analysis using real-world data from the JMDC Claims Database (JMDC Inc., Tokyo, Japan). This nationwide claims-based database includes health insurance claims data, medical examinations data, and ledger information received from Japanese health insurance associations contracted by JMDC since 2005; however, these are not national insurance data as patients in the database are employed people living in Japan and their dependents (22). Health insurance associations included in the database are associated with companies; self-employed workers and public servants and their families in Japan, who are members of other insurance associations, are not included in the database. As of November 2023, the cumulative database population size was approximately 17 million people (22). This study accessed data collected from 2005 to 2021.

A cross-sectional study was conducted on a yearly basis to investigate the prevalence and incidence of ADHD, the prevalence and risk ratios of psychiatric comorbidities in patients with ADHD, and the prevalence and risk ratio of ADHD in patients with specific psychiatric comorbidities. In the cross-sectional study, the total data collection period was from January 2017 to December 2021.

A retrospective cohort study, to enable longitudinal analysis, with an observation period of 2 years before and after the initial diagnosis of ADHD was used to investigate the order of diagnosis of psychiatric disorders in patients with ADHD. In the retrospective cohort study, the total data collection period was from January 2005 to December 2021. The enrollment period was from January 2017 to December 2019.

This study was conducted in accordance with the Guidelines for Good Pharmacoepidemiology Practice published by the International Society for Pharmacoepidemiology and local laws and regulations. This study was an observational study using anonymized information that had already been created by the JMDC; therefore, it was judged unnecessary to conduct ethical review, and informed consent was not required. The study was not registered.

### 2.2 Study population

The overall study population was male or female people living in Japan, aged 0–57 years, and who were registered in the JMDC database. The age cutoff was set to provide 10-year age categories for the adult population (18–27, 28–37, 38–47, and 48–57 years) and because there are very few people aged ≥60 years in the database. Study populations for both the cross-sectional and retrospective cohort analyses included people with a diagnosis of ADHD or other psychiatric disorders in the JMDC database, as described in detail below. In both analyses, patients with ADHD were defined in two ways. The first definition was by diagnosis: these patients had a diagnostic code for ADHD per the International Statistical Classification of Diseases and Related Health Problems 10th Revision (ICD-10) classification codes F90.x (hyperactivity disorder) and F98.8 (other specified behavioral and emotional disturbances that usually develop in children and adolescents) recorded in the database. Diagnostic codes for ADHD were selected in alignment with previous studies (17, 23–26). The second definition was by diagnosis and medication: these patients had a diagnostic code for ADHD and prescription code(s) for ADHD medication(s) approved in Japan recorded in the database (see **Supplementary Methods** for the Anatomical Therapeutic Chemical drug codes).

ADHD guidelines recommend that treatment of the more serious disorder should be prioritized in ADHD patients with psychiatric comorbidities (27, 28). ADHD is considered to predominate in patients prescribed ADHD medications. However, in cases where patients have concurrent psychiatric comorbidities, treatment of these other conditions may be prioritized, which can result in ADHD medications not being prescribed to patients with ADHD. Thus, patients with ADHD were defined in two ways to confirm whether a similar trend was seen in patients with predominant ADHD and those with predominant other psychiatric disorders.

#### 2.2.1 Cross-sectional study populations

In the cross-sectional study, five analysis populations were defined: overall, Population 1A, Population 1B, Population 2, and Population 3. The overall population consisted of patients who were continuously registered in the database for 12 months of the analysis year; analysis years were 2017–2021, and each analytical year was defined as January to December. For each analysis year, Population 1A included patients with a diagnosis of ADHD in that year; a patient could be enrolled for multiple years. The age of each patient was defined as their age as of April in each enrollment year. Patients must have had ADHD diagnosed at least once in the year of analysis; the index date was the month of initial diagnosis of ADHD in the year of analysis. Population 1B included patients with a diagnosis of ADHD and prescription of ADHD medication in each year. Patients must have had ADHD diagnosed and prescription of ADHD medication at least once in the year of analysis; the index date was the month of the first prescription of ADHD medication in the year of analysis. Population 2 included patients with a diagnosis of each psychiatric disorder in each year; a patient could be enrolled for multiple years and a patient could have more than one psychiatric disorder. Patients must have had a diagnosis of each psychiatric disorder at least once in the year of analysis; the index date was the month of initial diagnosis of each psychiatric disorder in the year of analysis (see **Supplementary Methods** for ICD-10 codes for psychiatric disorders). Population 3 included patients with no diagnosis of ADHD at baseline or during the entire period of registration. A baseline period was established for Population 3, defined as the preceding 2 years from the year of analysis; for example, if the year of analysis was 2021, 2019–2020 was the baseline period. If patients had been registered for ≥2 years, it was confirmed that they had no diagnosis of ADHD during the entire registered period.

Control populations were also defined. The Population 1A control (non-ADHD) population was defined as people continuously present in the database for 12 months in the year of analysis with no ADHD diagnosis in the year of analysis. The Population 1B control (non-ADHD) population was defined as people continuously present in the database for 12 months in the year of analysis without prescription of any ADHD medications in the year of analysis. The Population 2 control (no psychiatric disorders) populations were defined as people continuously present in the database for 12 months in the year of analysis and never diagnosed with each psychiatric disorder in the year of analysis.

#### 2.2.2 Retrospective cohort study populations

Cohort 1 consisted of patients with a diagnosis of ADHD. To meet the inclusion criteria for this cohort, patients were required to have ≥1 diagnosis of ADHD within the enrollment period and an established baseline period of the past 2 years including the index month. The index date was defined as the month of initial diagnosis of ADHD during the enrollment period. Patients must not have had a diagnosis of ADHD during the baseline period; if patients had been registered for ≥2 years, it was confirmed that they had no diagnosis of ADHD during the entire registered period. For children aged <2 years, the inclusion condition was that they could be followed up from birth, and the baseline period was defined as the period from birth to index.

Cohort 2 consisted of patients with a diagnosis of ADHD and prescribed ADHD medications, that is, patients from Cohort 1 who were prescribed ADHD medications at least once during the enrollment period in addition to their ADHD diagnosis. Included patients had an established baseline period of the past 2 years including the index month and must not have had a prescription of ADHD medication during the baseline period. If patients had been registered for ≥2 years, it was confirmed that they had no diagnosis of ADHD during the entire registered period. For Cohort 2 the index date was the month of the first prescription of ADHD medication during the enrollment period.

For both cohorts, a follow-up period was established, defined as 2 years from the month following the index month.

### 2.3 Variables/outcome measures

Patient demographic data included age (0–5 years, 6–11 years, 12–17 years, 18–27 years, 28–37 years, 38–47 years, 48–57 years), children and adolescents (0–17 years) or adults (18–57 years), and sex (male, female). Psychiatric disorders included substance use disorders; schizophrenia and schizotypal disorder; other psychotic disorders; mood disorders (including bipolar affective disorder, depressive episode, and recurrent depressive disorder); anxiety disorders; obsessive-compulsive disorder (OCD); reaction to severe stress, and adjustment disorders; dissociative disorders; somatoform disorders; eating disorders; intellectual disability (referred to as ‘mental retardation’ in the ICD-10); tic disorders; sleep disorders; ODD; conduct disorders (excluding ODD); specific developmental disorders (SDDs) of scholastic skills; SDD of motor function; ASD; and epilepsy.

This was a non-interventional study, and treatment was prescribed in accordance with current clinical practice at the discretion of the treating physician. ADHD medications data included prescriptions of ADHD medications approved in Japan; namely, osmotic-release oral system methylphenidate hydrochloride, lisdexamfetamine mesilate, atomoxetine hydrochloride, and guanfacine hydrochloride extended-release.

### 2.4 Endpoints

The cross-sectional study had several endpoints. The first endpoint was the annual prevalence and incidence of ADHD in Japan, in the overall population and Population 3, respectively. Absence of an ADHD diagnosis during the entire registered period was defined as a condition for new onset. Patients who had an established baseline period of the past ≥2 years were included in this study. The extended estimation for the Japanese population was calculated from the JMDC data after adjusting the number of patients in the JMDC database for age (in increments of 1 year) and sex (male, female) distribution in Japan using government statistics data (**Supplementary Methods**). The second endpoint was the prevalence and risk ratio of each psychiatric comorbidity in patients with ADHD (Population 1A and 1B) in each year. The control groups for risk ratio calculation were the Population 1A and 1B controls (non-ADHD populations), respectively, and the prevalence was calculated in the same manner as the ADHD population. The age and sex of the non-ADHD population were matched to those of the ADHD population to calculate the risk ratio (sample size ratio of 5:1). The risk ratio was calculated as “prevalence in the ADHD population/prevalence in the non-ADHD population.” The third endpoint was the prevalence and risk ratio of ADHD in patients with each psychiatric disorder (Population 2) in each year. The control group for risk ratio calculation constituted the Population 2 controls (no psychiatric disorders populations), and the prevalence was calculated in the same manner as the psychiatric disorder population. The age and sex of the no psychiatric disorders populations were matched to those of the psychiatric disorder population to calculate the risk ratio (sample size ratio of 2:1).

The endpoints for the retrospective cohort study were the prevalence of the first diagnosis of psychiatric disorders during the baseline (before initial ADHD diagnosis) and follow-up (after initial ADHD diagnosis) periods; the number of psychiatric disorders diagnosed during the baseline and follow-up periods (if the psychiatric disorder was diagnosed in both the baseline and follow-up periods, it was not counted in the follow-up period); and the time (months) from the index to the first diagnosis of each psychiatric disorder during the baseline and follow-up periods.

### 2.5 Statistical analysis

This cross-sectional study and cohort study used an existing database and are a descriptive epidemiological study without hypothesis verification; therefore, no sample size calculation was performed, and all patients who met the inclusion criteria were included. Categorical variables are summarized as number and proportion of patients.

In the cross-sectional study, the annual prevalence of ADHD in the JMDC database from 2017 to 2021 was calculated from the overall population; annual incidence of ADHD from 2017 to 2021 was calculated from Population 3. Estimates of ADHD prevalence and incidence in the overall Japanese population are shown for each year from 2017 to 2021. Annual prevalence and incidence of ADHD were calculated for the following subgroups: male children and adolescents (0–17 years), female children and adolescents (0–17 years), male adults (18–57 years), and female adults (18–57 years). For each of Populations 1A and 1B, and Population 1A and 1B controls (non-ADHD populations), the prevalence of each psychiatric comorbidity and risk ratio relative to the age- and sex-matched Population 1A and 1B controls (with 95% CI) were calculated by year from 2017 to 2021. For Population 2, the prevalence of ADHD and risk ratio relative to the age- and sex-matched Population 2 control (non-psychiatric comorbidity population) and 95% CI were calculated by year from 2017 to 2021. Although prevalence and risk ratios for Populations 1A, 1B, and 2 were calculated for each year from the JMDC database, only the data from 2019 are shown in this article; 2019 was selected as it precedes any potential mental health impact of the COVID-19 pandemic. Prevalence of psychiatric comorbidities among ADHD patients and prevalence of ADHD among psychiatric patients were calculated for the subgroups of children and adolescents (0–17 years) and adults (18–57 years).

In the longitudinal study, for Cohorts 1 and 2, the number and proportion of patients diagnosed with each psychiatric disorder during the baseline period or follow-up period were calculated (shown as percentage values only). The number of comorbid psychiatric disorders in the baseline period or follow-up period was calculated, together with summary statistics (mean, SD, minimum, Quartile 1 (Q1), median, Quartile 3 (Q3), and maximum). Summary statistics were also calculated for the number of months from the diagnosis of each psychiatric disorder to the index date (initial diagnosis of ADHD) during the baseline period and for the number of months from the index date to the initial diagnosis of each psychiatric disorder during the follow-up period.

No imputation was performed for missing values. The software programs used for statistical analysis were Amazon Redshift Version 1.0.47357 (Amazon Web Services, Seattle, WA, USA), SAS Version 9.4 (TS1M6) (SAS Institute Inc., Cary, NC, USA), and Python Version 3.11 (https://www.python.org/).

## 3 Results

### 3.1 Cross-sectional study patient disposition

**Figure 1:**
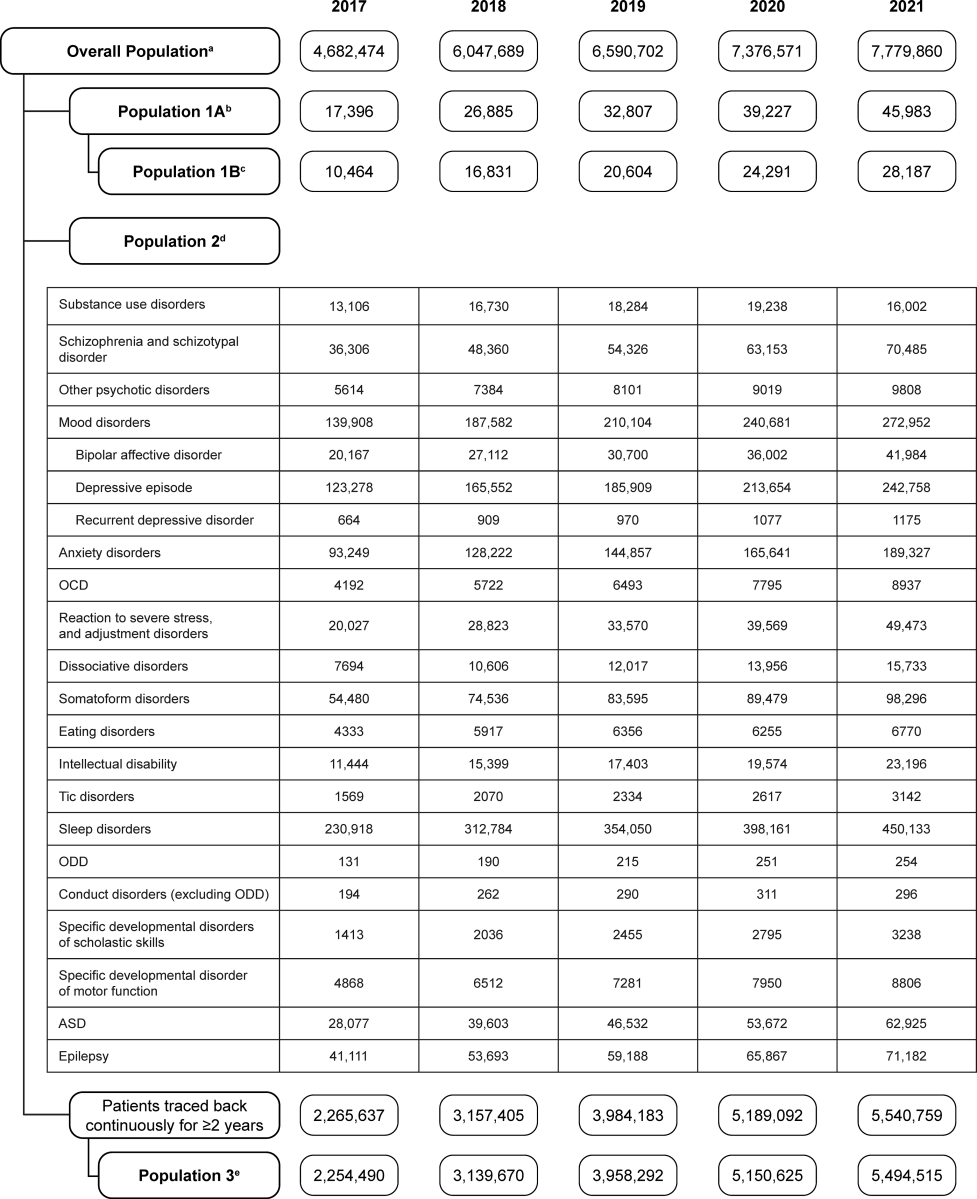
Patient flow diagram for the cross-sectional study, showing the number of patients in each population from 2017 to 2021. ^a^The overall population was the population who were continuously observed for 12 months in the year of the analysis. ^b^Population 1A was the population with a diagnosis of ADHD in each year. ^c^Population 1B was the population with a diagnosis of ADHD and prescription of ADHD medication in each year. ^d^Population 2 was the population with a diagnosis of a psychiatric comorbidity in each year. ^e^Population 3 was the population with no diagnosis of ADHD at baseline or during the entire period of registration. ADHD, attention deficit/hyperactivity disorder; ASD, autism spectrum disorder; OCD, obsessive-compulsive disorder; ODD, oppositional defiant disorder.

The full analysis population increased from 4,682,474 to 7,779,860 people from 2017 to 2021 (**Figure 1**). The number of patients with diagnosis codes of ADHD (Population 1A) increased from 17,396 to 45,983. The number of patients with a diagnosis of ADHD and prescription of ADHD drugs (Population 1B) increased from 10,464 to 28,187. The number of patients with a diagnosis of each psychiatric disorder (Population 2) also increased each year in most cases.

### 3.2 Cross-sectional study outcomes

#### 3.2.1 Annual prevalence and incidence of ADHD in Japan from 2017 to 2021

**Figure 2:**
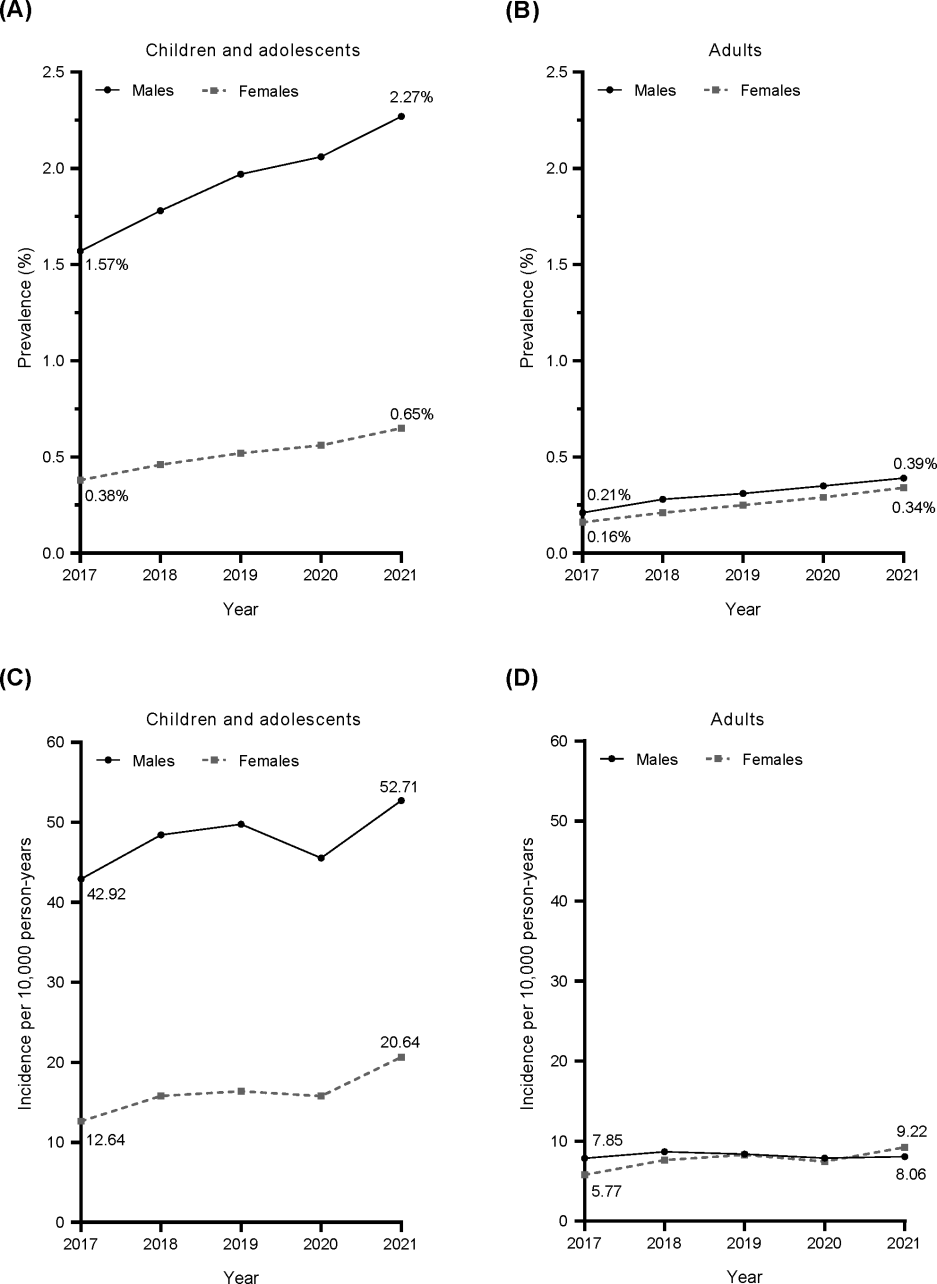
Annual prevalence **(A, B)** and incidence **(C, D)** of ADHD defined by diagnostic code (ICD-10 code) from 2017 to 2021. Circles and solid line: males; squares and dotted line: females. ADHD, attention deficit/hyperactivity disorder; ICD-10, International Statistical Classification of Diseases and Related Health Problems 10th Revision.

In children and adolescents, the estimated prevalence of ADHD in Japan increased from 2017 to 2021 in both boys and girls (**Figure 2A**). The prevalence of ADHD in boys was 3.5–4.1 times higher than that in girls. The prevalence of ADHD in adults also increased from 2017 to 2021, although it was lower than in children and adolescents, with only a small sex difference (**Figure 2B**). In 2021, the mean prevalence of ADHD in children and adolescents (both sexes combined) was estimated to be approximately 1.5%.

The estimated incidence of ADHD in children and adolescents in Japan increased from 2017 to 2021 in both boys and girls, except for a temporary decline in 2020 (**Figure 2C**). The incidence of ADHD in boys was 2.6–3.4 times higher than that in girls. ADHD incidence in adults was lower than in children, with a small difference between males and females (**Figure 2D**). While the incidence of ADHD among male adults did not show any clear changes, the incidence of ADHD among female adults slightly but steadily increased (except in 2020). Similar trends were observed for the estimated annual prevalence and annual incidence of ADHD in Japan when estimated from Population 1B data (**Supplementary Figure 1**).

#### 3.2.2 Twelve-month psychiatric comorbidities of ADHD

In 2019, children and adolescents with ADHD in the JMDC database had a higher prevalence of all psychiatric disorders tested than the control (non-ADHD) population (**Table 1**). The most common comorbidity in children and adolescents with ADHD was ASD (54.4%). Other common comorbidities (prevalence ≥5%) in children and adolescents with ADHD were sleep disorders (13.7%); schizophrenia and schizotypal disorder (13.1%); mood disorders (9.1%; including depressive episode 6.3%); intellectual disability (8.7%); somatoform disorders (8.7%); reaction to severe stress, and adjustment disorders (7.7%); anxiety disorders (6.9%); epilepsy (6.1%); and SDDs of scholastic skills (5.0%). Children and adolescents with ADHD had risk ratios >1 for all psychiatric disorders tested except recurrent depressive disorder (risk ratio could not be calculated). Risk ratios were high (≥25) for ODD (111.4), conduct disorders excluding ODD (56.1), schizophrenia and schizotypal disorder (44.5), SDDs of scholastic skills (41.3), other psychotic disorders (38.6), bipolar affective disorder (36.4), and ASD (26.4). The lowest risk ratio observed was for substance use disorders (2.9). When ADHD was defined by prescriptions for ADHD medications in addition to diagnostic codes, risk ratios for many psychiatric disorders tended to decrease slightly (**Supplementary Table 1**). The reduction in risk ratio was more pronounced for externalizing disorders, SDDs of scholastic skills, and other psychotic disorders.

**Table 1:** Twelve-month psychiatric comorbidities of ADHD^a^ in 2019.

Psychiatric disorder Disorder subclassification	Children and adolescents			Adults		
	With ADHD (Population 1A) N = 20,220	Without ADHD (Population 1A control)^b^ N = 101,100	Risk ratio (95% CI)	With ADHD (Population 1A) N = 12,416	Without ADHD (Population 1A control)^b^ N = 62,080	Risk ratio (95% CI)
Substance use disorders	7 (<0.1)	12 (<0.1)	2.9 (1.1–7.4)	208 (1.7)	228 (0.4)	4.6 (3.8–5.5)
Schizophrenia and schizotypal disorder	2642 (13.1)	297 (0.3)	44.5 (39.5–50.1)	2872 (23.1)	540 (0.9)	26.6 (24.3–29.1)
Other psychotic disorders	255 (1.3)	33 (<0.1)	38.6 (26.9–55.5)	582 (4.7)	77 (0.1)	37.8 (29.8–47.9)
Mood disorders	1843 (9.1)	416 (0.4)	22.2 (19.9–24.6)	7559 (60.9)	2078 (3.3)	18.2 (17.4–19.0)
Bipolar affective disorder	481 (2.4)	66 (0.1)	36.4 (28.2–47.1)	2320 (18.7)	314 (0.5)	36.9 (32.9–41.5)
Depressive episode	1272 (6.3)	313 (0.3)	20.3 (18.0–23.0)	6436 (51.8)	1815 (2.9)	17.7 (16.9–18.6)
Recurrent depressive disorder	1 (<0.1)	0 (0.0)	–	41 (0.3)	8 (<0.1)	25.6 (12.0–54.6)
Anxiety disorders	1392 (6.9)	558 (0.6)	12.5 (11.3–13.7)	2803 (22.6)	1274 (2.1)	11.0 (10.3–11.7)
OCD	184 (0.9)	58 (0.1)	15.9 (11.8–21.3)	277 (2.2)	75 (0.1)	18.5 (14.3–23.8)
Reaction to severe stress, and adjustment disorders	1559 (7.7)	455 (0.4)	17.1 (15.4–19.0)	1419 (11.4)	375 (0.6)	18.9 (16.9–21.2)
Dissociative disorders	108 (0.5)	41 (<0.1)	13.2 (9.2–18.69)	496 (4.0)	98 (0.2)	25.3 (20.4–31.4)
Somatoform disorders	1761 (8.7)	1024 (1.0)	8.6 (8.0–9.3)	927 (7.5)	657 (1.1)	7.1 (6.4–7.8)
Eating disorders	81 (0.4)	100 (0.1)	4.1 (3.0–5.4)	114 (0.9)	73 (0.1)	7.8 (5.8–10.5)
Intellectual disability	1760 (8.7)	770 (0.8)	11.4 (10.5–12.4)	403 (3.2)	151 (0.2)	13.3 (11.1–16.1)
Tic disorders	342 (1.7)	159 (0.2)	10.8 (8.9–13.0)	40 (0.3)	2 (<0.1)	100.0 (24.2–413.7)
Sleep disorders	2777 (13.7)	904 (0.9)	15.4 (14.3–16.5)	6012 (48.4)	2666 (4.3)	11.3 (10.8–11.8)
ODD	156 (0.8)	7 (<0.1)	111.4 (52.3–237.6)	9 (0.1)	0 (0.0)	–
Conduct disorders (excluding ODD)	101 (0.5)	9 (<0.1)	56.1 (28.4–110.9)	20 (0.2)	2 (<0.1)	50.0 (11.7–213.9)
SDDs of scholastic skills	1017 (5.0)	123 (0.1)	41.3 (34.3–49.8)	112 (0.9)	6 (<0.1)	93.3 (41.1–212.1)
SDD of motor function	479 (2.4)	315 (0.3)	7.6 (6.6–8.8)	22 (0.2)	12 (<0.1)	9.2 (4.5–18.5)
ASD	11,003 (54.4)	2082 (2.1)	26.4 (25.3–27.6)	2728 (22.0)	226 (0.4)	60.4 (52.8–69.0)
Epilepsy	1243 (6.1)	873 (0.9)	7.1 (6.5–7.8)	1425 (11.5)	516 (0.8)	13.8 (12.5–15.2)

All data are n (prevalence %) unless otherwise stated. ^a^ADHD was defined by ICD-10 diagnostic codes (F90 and F98.8). ^b^Non-ADHD population, age- and sex-matched to Population 1A (sample size ratio of 5:1). ADHD, attention deficit/hyperactivity disorder; ASD, autism spectrum disorder; ICD-10, International Statistical Classification of Diseases and Related Health Problems 10th Revision; OCD, obsessive-compulsive disorder; ODD, oppositional defiant disorder; SDD, specific developmental disorder.

In 2019, adult patients with ADHD also had a higher prevalence of all psychiatric disorders tested than the control population (**Table 1**). The most common comorbidity in adult patients with ADHD was mood disorders (60.9%; including depressive episode [51.8%] and bipolar affective disorder [18.7%]). Other common comorbidities (prevalence ≥5%) in adults with ADHD were sleep disorders (48.4%); schizophrenia and schizotypal disorder (23.1%); anxiety disorders (22.6%); ASD (22.0%); epilepsy (11.5%); reaction to severe stress, and adjustment disorder (11.4%); and somatoform disorders (7.5%). Adult patients with ADHD had risk ratios >1 for all psychiatric disorders tested except ODD (risk ratio could not be calculated). In particular, the risk ratios were high (≥25) for patients with tic disorders (100.0), SDDs of scholastic skills (93.3), ASD (60.4), conduct disorders excluding ODD (50.0), other psychotic disorders (37.8), bipolar affective disorder (36.9), schizophrenia and schizotypal disorder (26.6), recurrent depressive disorder (25.6), and dissociative disorders (25.3). The lowest risk ratio observed was for substance use disorders (4.6). When ADHD was defined by prescriptions for ADHD medications in addition to diagnostic codes, risk ratios for many psychiatric disorders tended to decrease slightly (**Supplementary Table 1**). The reduction in risk ratio was more pronounced for conduct disorders, recurrent depressive disorder, eating disorders, tic disorders, and other neurodevelopmental disorders such as ASD, SDDs of scholastic skills, SDD of motor function, and intellectual disability.

There was no obvious change in the prevalence patterns of most of the 12-month psychiatric comorbidities of ADHD in children/adolescents and adults observed from 2017 to 2021 (only 2019 data shown). However, the prevalence of ASD among children/adolescents with ADHD tended to increase over time (51.2% in 2017 to 56.1% in 2021), while the risk ratios tended to decrease (27.3 in 2017 to 24.2 in 2021). The prevalence of mood disorders among adults with ADHD did not change over time (62.1% in 2017 to 61.2% in 2021), while the risk ratios tended to decrease (21.1 in 2017 to 16.5 in 2021).

#### 3.2.3 ADHD as a 12-month comorbidity of various psychiatric disorders

In 2019, the prevalence of ADHD as a comorbidity in child and adolescent patients with psychiatric disorders in the JMDC database (Population 2) was generally higher than in each Population 2 control group (**Table 2**). ADHD prevalence was highest (≥20%) among patients with ODD (77.2%); conduct disorders excluding ODD (49.3%); SDDs of scholastic skills (48.6%); schizophrenia and schizotypal disorder (41.3%); bipolar affective disorder (35.7%); other psychotic disorders (34.2%); ASD (30.8%); mood disorders (22.5%); and reaction to severe stress, and adjustment disorders (21.5%). The risk ratio for having ADHD was >1 for all psychiatric diseases in children and adolescents, except for those with recurrent depressive disorder (risk ratio could not be calculated). Risk ratios for ADHD were highest (≥25.0) among patients with ODD (44.6), ASD (35.4), conduct disorders excluding ODD (33.7), bipolar affective disorder (30.1), schizophrenia and schizotypal disorder (29.7), and SDDs of scholastic skills (27.1).

**Table 2:** ADHD^a^ as a 12-month comorbidity of various psychiatric disorders in 2019.

Psychiatric disorder Disorder subclassification	Children and adolescents					Adults				
	With each psychiatric disorder (Population 2)		Without each psychiatric disorder (Population 2 control)^b^		Risk ratio (95% CI)	With each psychiatric disorder (Population 2)		Without each psychiatric disorder (Population 2 control)^b^		Risk ratio (95% CI)
	Population N	ADHD prevalence, n (%)	Population N	ADHD prevalence, n (%)		Population N	ADHD prevalence, n (%)	Population N	ADHD prevalence, n (%)	
Substance use disorders	271	7 (2.6)	542	2 (0.4)	7.0 (1.5–33.5)	15,510	208 (1.3)	31,020	82 (0.3)	5.1 (3.9–6.5)
Schizophrenia and schizotypal disorder	6395	2642 (41.3)	12,790	178 (1.4)	29.7 (25.6–34.4)	42,911	2872 (6.7)	85,822	182 (0.2)	31.6 27.2–36.6)
Other psychotic disorders	745	255 (34.2)	1490	22 (1.5)	23.2 (15.1–35.5)	6504	582 (8.9)	13,008	38 (0.3)	30.6 (22.1–42.5)
Mood disorders	8173	1843 (22.5)	16,346	169 (1.0)	21.8 (18.7–25.5)	178,468	7559 (4.2)	356,936	320 (0.1)	47.2 (42.2–52.8)
Bipolar affective disorder	1348	481 (35.7)	2696	32 (1.2)	30.1 (21.1–42.7)	26,636	2320 (8.7)	53,272	105 (0.2)	44.2 (36.4–53.7)
Depressive episode	6421	1272 (19.8)	12,842	119 (0.9)	21.4 (17.8–25.7)	158,694	6436 (4.1)	317,388	362 (0.1)	35.6 (32.0–39.5)
Recurrent depressive disorder	6	1 (16.7)	12	0 (0.0)	–	843	41 (4.9)	1686	2 (0.1)	41.0 (9.9–169.1)
Anxiety disorders	9399	1392 (14.8)	18,798	217 (1.2)	12.8 (11.1–14.8)	113,233	2803 (2.5)	226,466	410 (0.2)	13.7 (12.3–15.2)
OCD	944	184 (19.5)	1888	23 (1.2)	16.0 (10.4–24.5)	5198	277 (5.3)	10,396	28 (0.3)	19.8 (13.4–29.1)
Reaction to severe stress, and adjustment disorder	7268	1559 (21.5)	14,536	205 (1.4)	15.2 (13.2–17.5)	25,096	1419 (5.7)	50,192	140 (0.3)	20.3 (17.1–24.1)
Dissociative disorders	1064	108 (10.2)	2128	20 (0.9)	10.8 (6.7–17.3)	9475	496 (5.2)	18,950	46 (0.2)	21.6 (16.0–29.1)
Somatoform disorders	15,545	1761 (11.3)	31,090	414 (1.3)	8.5 (7.7–9.5)	56,366	927 (1.6)	112,732	231 (0.2)	8.0 (7.0–9.3)
Eating disorders	2006	81 (4.0)	4012	36 (0.9)	4.5 (3.1–6.6)	3860	114 (3.0)	7720	26 (0.3)	8.8 (5.7–13.4)
Intellectual disability	11,458	1760 (15.4)	22,916	300 (1.3)	11.7 (10.4–13.2)	5880	403 (6.9)	11,760	56 (0.5)	14.4 (10.9–19.0)
Tic disorders	2028	342 (16.9)	4056	69 (1.7)	9.9 (7.7–12.8)	286	40 (14.0)	572	1 (0.2)	80.0 (11.1–579.0)
Sleep disorders	16,802	2777 (16.5)	33,604	375 (1.1)	14.8 (13.3–16.5)	257,711	6012 (2.3)	515,422	513 (0.1)	23.4 (21.4–25.6)
ODD	202	156 (77.2)	404	7 (1.7)	44.6 (21.3–93.2)	13	9 (69.2)	26	0 (0.0)	–
Conduct disorders (excluding ODD)	205	101 (49.3)	410	6 (1.5)	33.7 (15.0–75.4)	83	20 (24.1)	166	0 (0.0)	–
SDDs of scholastic skills	2093	1017 (48.6)	4186	75 (1.8)	27.1 (21.6–34.1)	350	112 (32.0)	700	1 (0.1)	224.0 (31.4–1597.5)
SDD of motor function	6955	479 (6.9)	13,910	122 (0.9)	7.9 (6.5–9.6)	325	22 (6.8)	650	0 (0.0)	–
ASD	35,756	11,003 (30.8)	71,512	621 (0.9)	35.4 (32.7–38.4)	10,670	2728 (25.6)	21,340	77 (0.4)	70.9 (56.6–88.8)
Epilepsy	13,543	1243 (9.2)	27,086	341 (1.3)	7.3 (6.5–8.2)	38,769	1425 (3.7)	77,538	194 (0.3)	14.7 (12.7–17.1)

All data are n (prevalence %) unless otherwise stated. ^a^ADHD was defined by ICD-10 diagnostic codes (F90 and F98.8). ^b^Age- and sex-matched to Population 2 (sample size ratio of 2:1). ADHD, attention deficit/hyperactivity disorder; ASD, autism spectrum disorder; ICD-10, International Statistical Classification of Diseases and Related Health Problems 10th Revision; OCD, obsessive-compulsive disorder; ODD, oppositional defiant disorder; SDD, specific developmental disorder.

In 2019, the prevalence of ADHD was also higher in adult patients with various psychiatric disorders than in each Population 2 control group (**Table 2**). The highest prevalence (≥20%) of adult ADHD was found in patients with ODD (69.2%), SDDs of scholastic skills (32.0%), ASD (25.6%), and conduct disorders excluding ODD (24.1%). The risk ratio for having ADHD was >1 for all psychiatric diseases in adults, except for those with ODD, conduct disorders excluding ODD, and SDD of motor function (risk ratios could not be calculated). Psychiatric disorders with a high risk of comorbid ADHD (risk ratio ≥25.0) in adults included SDDs of scholastic skills (224.0), tic disorders (80.0), ASD (70.9), mood disorders (47.2), bipolar affective disorder (44.2), recurrent depressive disorder (41.0), depressive episode (35.6), schizophrenia and schizotypal disorder (31.6), and other psychotic disorders (30.6).

When the presence of ADHD was defined by prescriptions for ADHD medications in addition to diagnostic codes, the risk ratio for ADHD was slightly increased for children and adolescents with many psychiatric disorders (**Supplementary Table 2**). The risk ratio for ADHD remained unchanged for adults with most psychiatric disorders, slightly increased for adults with bipolar affective disorder and recurrent depressive disorder, and decreased for adults with eating disorders, intellectual disability, tic disorders, SDDs of scholastic skills, and ASD.

There was no obvious change in the trend of 12-month ADHD prevalence among psychiatric comorbidities observed from 2017 to 2021 in both children/adolescents and adults (only 2019 data shown).

### 3.3 Longitudinal study outcomes

#### 3.3.1 Retrospective cohort study patient disposition

**Figure 3:**
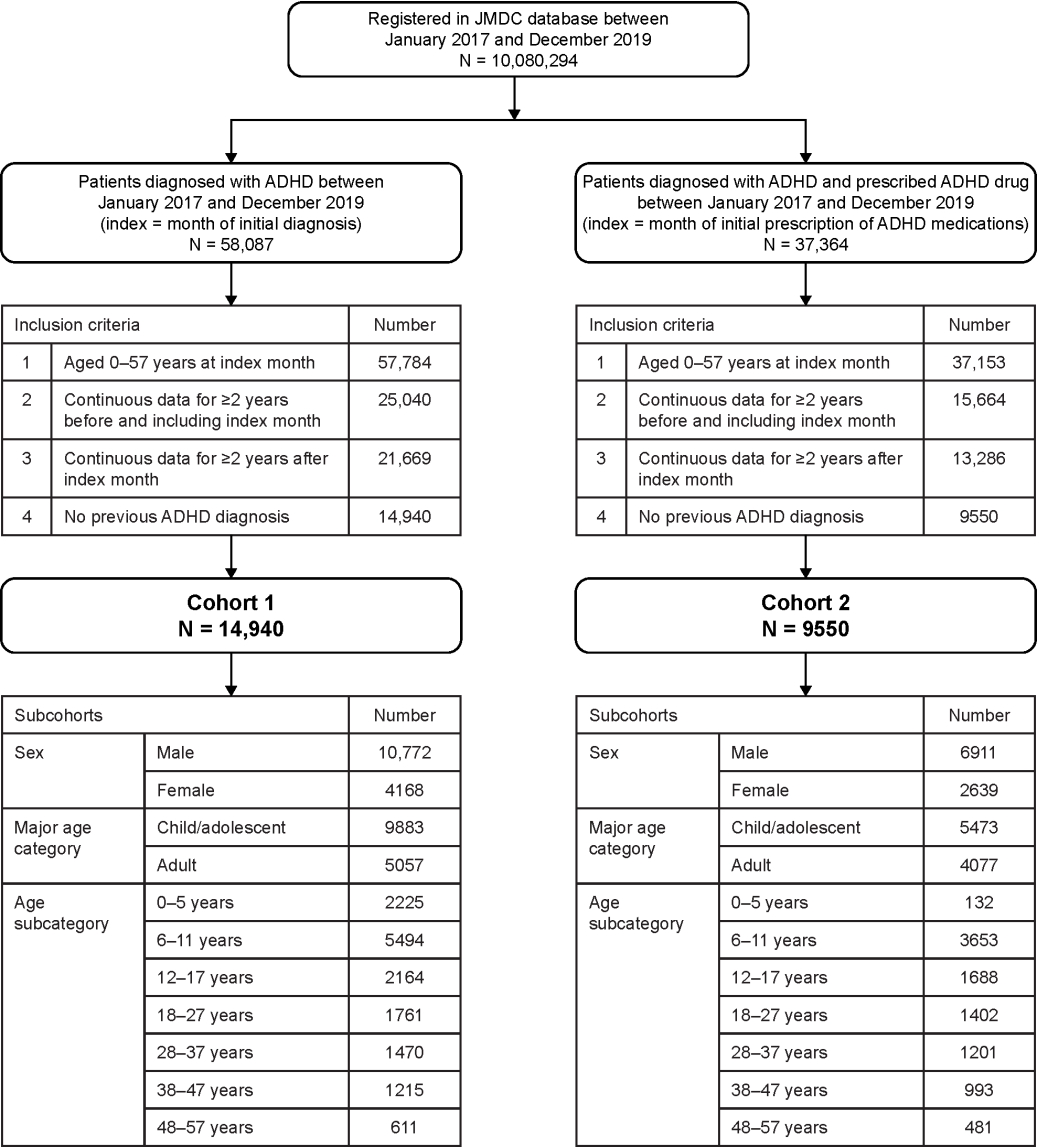
Patient flow diagram for the retrospective cohort study (longitudinal study). ADHD, attention deficit/hyperactivity disorder.

There were 10,080,294 people registered in the JMDC database between January 2017 and December 2019 (**Figure 3**). There were 58,087 patients with an ADHD diagnosis, and 37,364 patients with an ADHD diagnosis and prescribed ADHD medications. Among patients diagnosed with ADHD, the number of patients included in Cohort 1 was 14,940. Among patients diagnosed with ADHD and receiving ADHD medication, the number of patients included in Cohort 2 was 9550. The proportion of patients in each age subcategory was generally similar between the cohorts, with the exception of patients aged 0–5 years, who comprised 1.4% of Cohort 2 versus 14.9% of Cohort 1.

#### 3.3.2 Psychiatric disorders diagnosed before and after initial ADHD diagnosis

**Figure 4:**
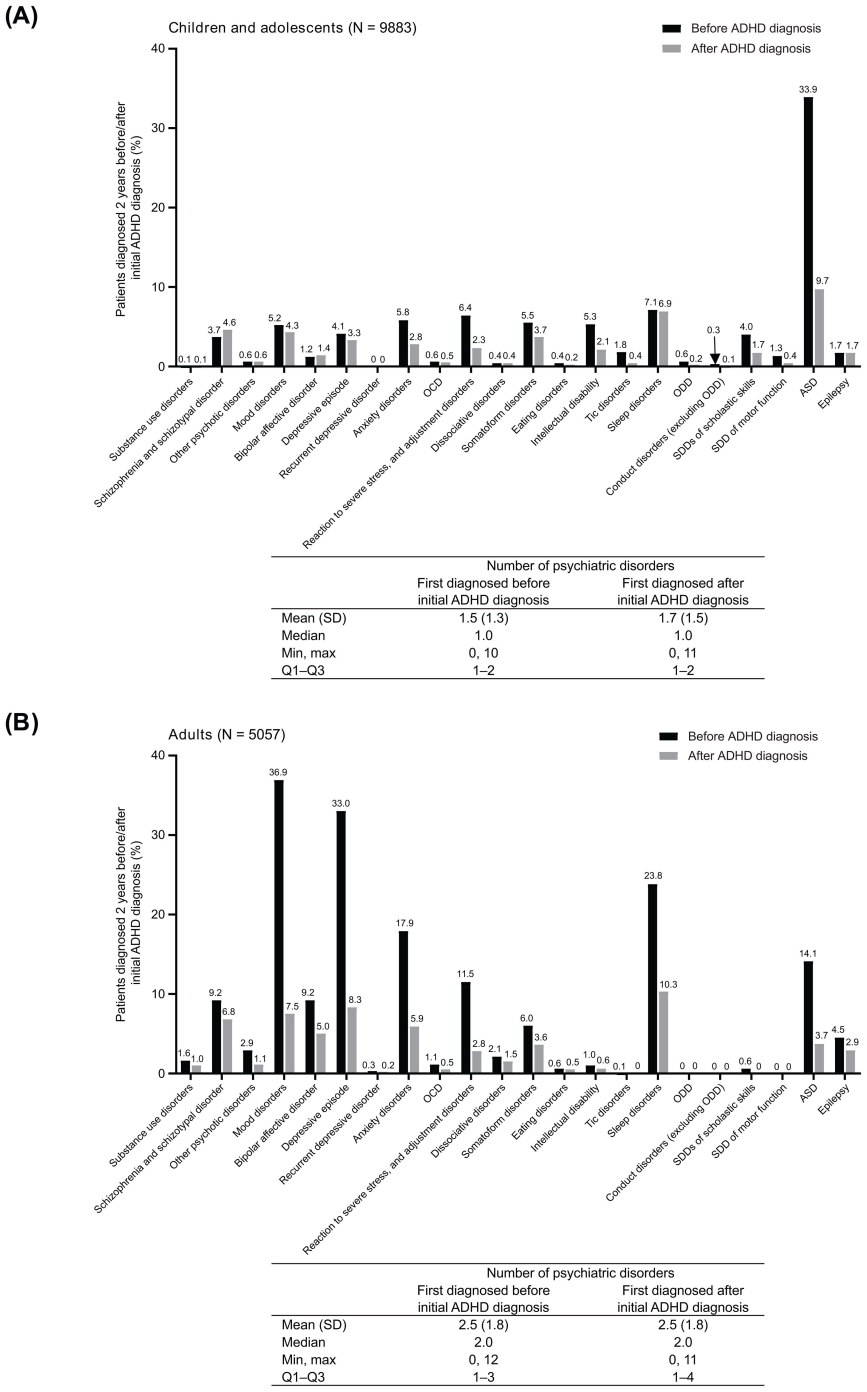
Percentage of child and adolescent patients **(A)** and adult patients **(B)** with ADHD who were first diagnosed with a psychiatric disorder 2 years before (black columns) or after (grey columns) the initial diagnosis of ADHD. Tables show summary statistics for the number of psychiatric disorders diagnosed before and after the initial ADHD diagnosis. ADHD was defined by ICD-10 diagnostic codes (F90 and F98.8). ADHD, attention deficit/hyperactivity disorder; ASD, autism spectrum disorder; ICD-10, International Statistical Classification of Diseases and Related Health Problems 10th Revision; max, maximum; min, minimum; OCD, obsessive-compulsive disorder; ODD, oppositional defiant disorder; SDD, specific developmental disorder; Q, Quartile.

In children and adolescents with ADHD, the psychiatric disorder most commonly diagnosed prior to diagnosis of ADHD was ASD (33.9%) (**Figure 4A**). Other psychiatric disorders diagnosed prior to the initial diagnosis of ADHD in ≥5% of patients were sleep disorders (7.1%); reaction to severe stress, and adjustment disorders (6.4%); anxiety disorders (5.8%); somatoform disorders (5.5%); intellectual disability (5.3%); and mood disorders (5.2%). The median number of psychiatric disorders diagnosed before the initial ADHD diagnosis in child and adolescent ADHD patients was 1 (Q1–Q3: 1–2). The median number of psychiatric disorders diagnosed after the initial ADHD diagnosis was also 1 (Q1–Q3: 1–2). ASD was substantially more likely to be diagnosed before ADHD (33.9% of patients diagnosed within 2 years before ADHD diagnosis) than after (9.7% of patients diagnosed within 2 years after ADHD diagnosis) (**Figure 4A**). Other psychiatric disorders more likely to be first diagnosed before than after the initial ADHD diagnosis included mood disorders (including depressive episodes); anxiety disorders; reaction to severe stress, and adjustment disorders; somatoform disorders; intellectual disability; SDDs of scholastic skills; and SDD of motor function.

In adults with ADHD, the most prevalent psychiatric disorder prior to the initial diagnosis of ADHD was mood disorders (36.9%; including depressive episode [33.0%] and bipolar affective disorder [9.2%]) (**Figure 4B**). Other psychiatric disorders diagnosed prior to the initial diagnosis of ADHD in ≥5% of patients were sleep disorders (23.8%); anxiety disorders (17.9%); ASD (14.1%); reaction to severe stress, and adjustment disorders (11.5%); schizophrenia and schizotypal disorder (9.2%); and somatoform disorders (6.0%). In adult patients with ADHD, the median number of psychiatric disorders diagnosed before the initial ADHD diagnosis was 2 (Q1–Q3: 1–3); the median number of psychiatric disorders diagnosed after the initial ADHD diagnosis was also 2 (Q1–Q3: 1–4). In adults, the psychiatric disorders that were more commonly diagnosed before than after the initial diagnosis of ADHD included substance use disorders; schizophrenia and schizotypal disorder; other psychotic disorders; mood disorders (including bipolar affective disorder, depressive episode, and recurrent depressive disorder); anxiety disorders; OCD; reaction to severe stress, and adjustment disorders; dissociative disorders; somatoform disorders; intellectual disability; sleep disorders; ASD; and epilepsy (**Figure 4B**).

Common psychiatric disorders diagnosed before and after the initial diagnosis of ADHD were similar when ADHD was defined by prescribing ADHD medications in addition to the ADHD diagnosis for both children/adolescents and adults (**Supplementary Figure 2**).

The intervals between diagnoses of ADHD and diagnoses of other psychiatric disorders are shown in **Supplementary Figure 3** for both children and adolescents (**Supplementary Figures 3A, B**) and adults (**Supplementary Figures 3C, D**). When other psychiatric disorders were diagnosed before ADHD, a median interval ≤2 months was observed between a diagnosis of other psychotic disorders; mood disorders; depressive episode; anxiety disorders; reaction to severe stress, and adjustment disorders; intellectual disability; tic disorders; SDDs of scholastic skills; and ASD, and a diagnosis of ADHD in both children and adolescents (**Supplementary Figure 3A**) and in adults (**Supplementary Figure 3C**). The median time from a diagnosis of substance use disorders, schizophrenia and schizotypal disorder, OCD, dissociative disorders, somatoform disorders, eating disorders, sleep disorders, SDD of motor function, and epilepsy to a diagnosis of ADHD was 3–7 months in children and adolescents and 3–8 months in adults. In some cases, the diagnosis of ADHD took up to 2 years after the diagnosis of other psychiatric disorders. When ADHD was diagnosed before other psychiatric disorders, the median interval before other psychiatric disorders were diagnosed in children and adolescents was 4–16 months (**Supplementary Figure 3B**). In adults, median intervals between ADHD diagnosis and diagnosis of other psychiatric disorders were 6–15 months (**Supplementary Figure 3D**). When ADHD was defined by prescribing ADHD medications in addition to the ADHD diagnosis, there was an overall slight increase in the intervals between diagnoses for both children/adolescents and adults (**Supplementary Figure 4**).

## 4 Discussion

This nationwide population-based real-world study demonstrated that there is a high risk of ADHD being comorbid with psychiatric disorders in children and adolescents and in adults in Japan. This study also demonstrated that ASD was commonly diagnosed prior to the initial diagnosis of ADHD in children and adolescents, and various psychiatric disorders were diagnosed prior to the initial diagnosis of ADHD in adults. The primary clinical implication of this study is that it is important to carefully assess the likelihood of comorbid ADHD when diagnosing patients with psychiatric disorders.

In this claims-based database study, the prevalence of ADHD among children and adolescents in 2017 (1.6% in males and 0.4% in females) was higher than reported in previous Japanese database studies (13, 14); prevalence then increased from 2017 to 2021. It is likely that improved awareness of ADHD has led to increased diagnosis, and thus more recent studies may have higher estimates than older studies, and prevalence is likely to increase over time. The maximum prevalence estimated for children and adolescents in this study was 2.3% in male children and 0.6% in female children in 2021. Both estimates were lower than those obtained from an epidemiological survey conducted in municipal areas of Japan in 2009–2011 (5.8%) (12), US counties in 2016–2018 (12.9%) (29), and provinces of Canada in 1996–2016 (2.6–8.6%) (30). The nationwide estimate of ADHD prevalence in adults in this study was 0.3–0.4%, similar to a database-based estimate of prevalence in male adults in the UK (0.3%) (31), but lower than an epidemiological survey estimate of prevalence in the Japanese city of Hamamatsu (1.65%) (15). These differences in prevalence estimates are most likely primarily a result of differences in study design (claims-based database studies vs epidemiological surveys). Previous epidemiological surveys likely show a more accurate estimate of the prevalence of ADHD in the general population as they are based on screening, identification, and assessment of people with ADHD traits who fit the diagnostic criteria, albeit within a limited geographic area. The current claims-based study only included people diagnosed by a physician at a medical institution, so it is unsurprising that the estimated prevalence rate is lower than in previous epidemiological surveys in Japan. The lower prevalence in the current study suggests that there may be a population of people with ADHD who have not been diagnosed and therefore were not detected in our database study. In our database study, the mean prevalence of ADHD in children and adolescents (both sexes combined) was estimated to be about 1.5%, which is approximately one-quarter of 5.8%, the ADHD prevalence estimated from a previous epidemiological survey (12).

In this study, from 2017 to 2021, the incidence of ADHD in children and adolescents increased from 42.92 to 52.71 per 10,000 person-years in males and from 12.64 to 20.64 per 10,000 person-years in females. These results are generally consistent, during the overlapping time periods, with a previous Japanese national database study between 2010 and 2019 (24). However, the previous study by Sasayama et al. showed that the incidence of ADHD in Japan slowed from 2018 to 2019, after a robust increase in the preceding years (24), while our results showed an increase in the incidence of ADHD for both male and female children and adolescents from 2017 to 2021 (except for a transient decrease in 2020). In adult females in the present study, there was a small increase in incidence from 5.77 to 9.22 per 10,000 person-years from 2017 to 2021. However, in adult males in the present study, the annual incidence was ~8 per 10,000 person-years, with no major change in incidence from 2017 to 2021. Nonetheless, there was a noticeable decrease in incidence for both males and females in 2020, which could be attributed to a reluctance to seek hospital visits amid the spread of the COVID-19 pandemic (32). The annual incidences of ADHD in Japanese children/adolescents and adults in our study were higher than those reported from a UK database study in 2000–2018 (31). National differences in the incidence of ADHD are most likely influenced by differences in both culture and healthcare systems.

In this study, patients with ADHD had a high risk of psychiatric comorbidities, and patients with other psychiatric disorders had a high risk of comorbid ADHD. This was the case for both children/adolescents and adults, and a very similar pattern was seen in each year from 2017 to 2021 (only 2019 data shown). In 2019, which was selected because it preceded any potential mental health impact of the COVID-19 pandemic, the 12-month psychiatric comorbidities of ADHD in children with the highest risk ratios were externalizing disorders (ODD, conduct disorders), SDDs of scholastic skills, schizophrenia and schizotypal disorder, other psychotic disorders, bipolar affective disorder, and ASD. When comorbidity of ADHD was examined in children and adolescents with various psychiatric disorders, the risk ratio for ADHD was highest in patients with externalizing disorders, ASD, bipolar affective disorder, schizophrenia and schizotypal disorder, and SDDs of scholastic skills. These results are generally consistent with previous reports of common psychiatric comorbidities of ADHD in childhood, particularly the comorbidity of ADHD with ASD (16, 17, 33).

In adults with ADHD, the prevalence of neurodevelopmental disorders and externalizing disorders was lower than that in children, and the prevalence of internalizing disorders (for example, schizophrenia and schizotypal disorder, mood disorders, and anxiety disorders) was higher than that in children and adolescents. In particular, the prevalence of both mood disorders, including bipolar affective disorder and depressive episodes, and sleep disorders was higher than that in children and adolescents. Risk ratios for neurodevelopmental disorders such as ASD and tic disorders were higher in adults with ADHD than in child and adolescent patients, probably because the prevalence in the adult control population was lower than in the control population for children and adolescents. Similarly, risk ratios for mood disorders and psychotic disorders were lower in adults with ADHD than in children and adolescents, probably because the prevalence in the adult control population was higher than in the child and adolescent control population. In adults with psychiatric disorders, the prevalence of comorbid ADHD was lower than that in children and adolescents for almost all psychiatric disorders. For adults with ASD, prevalence of comorbid ADHD was only slightly lower than in children and adolescents (25.6% vs 30.8%, respectively). However, the risk ratio of ADHD for adults with ASD was higher than that for children and adolescents, probably because of the lower prevalence of comorbid ADHD in the adult control population than in the child and adolescent control population.

The prevalence of psychiatric disorders observed as 12-month comorbidities of ADHD in adults in Japan were broadly consistent with previous reports of 12-month comorbidities of ADHD in adults in other countries (9, 18). However, the prevalence of mood disorders was higher and the prevalence of substance use disorders was lower in Japanese adults with ADHD than reported in other countries (9, 18). In the present study, in many Japanese adults with ADHD, mood disorders were diagnosed before ADHD, suggesting that mood disorder symptoms may prompt patients to visit a hospital and subsequently be diagnosed with ADHD. In addition, public awareness of depression is relatively high in Japan as the mental health of employees is regularly screened under the Government’s Stress Check Program (34), and there is easily accessible public health education about depression. These features of Japanese society and health care may partly explain why the prevalence of mood disorders, especially depressive episodes, was higher in our database study than in overseas epidemiological studies. By contrast, the prevalence of substance use disorders is known to be generally lower in Japan than in Western countries (35). In addition, substance use disorders in Japan associated with the abuse or overdose of over-the-counter (OTC) drugs are increasing, and this form of substance use disorders may not be as readily captured in claims databases (36). In addition, it appears that abuse of OTC drugs in Japan is more strongly associated with mood disorders and neurotic, stress-related, and somatoform disorders than with behavioral and emotional disorders, including ADHD (36).

In the present study, we also examined the order of diagnosis of ADHD and other psychiatric conditions. In Japanese children, the most common psychiatric diagnosis prior to a diagnosis of ADHD was ASD. Symptoms of ASD are often noted before symptoms of ADHD, and incidence of ASD tends to peak at an earlier age (23). ASD is often diagnosed by 3 years of age, whereas ADHD in children may be more frequently identified and diagnosed in school-aged children, often when children are facing challenges in school. Adults with ADHD in the present study had a larger range of psychiatric diseases diagnosed in the 2 years prior to ADHD diagnosis than did children. The psychiatric disorders diagnosed in ≥10% of patients before the diagnosis of ADHD in adults included mood disorders, including depressive episodes; anxiety disorders; reaction to severe stress, and adjustment disorders; sleep disorders; and ASD. This is somewhat consistent with a previous study from Spain, in which mood disorders and anxiety disorders were commonly diagnosed before ADHD; however, substance use disorders were the most commonly diagnosed disorder prior to ADHD in the Spanish population (37). If the diagnosis of another psychiatric disorder precedes the diagnosis of ADHD, ADHD symptoms or problems in daily life may not have improved despite treatment of the other psychiatric disorder, leading to the subsequent diagnosis of ADHD. It is therefore important to carefully assess the likelihood of comorbid ADHD when diagnosing adult patients with psychiatric disorders.

In the present study, ADHD was defined in two ways: by diagnostic codes only, and by diagnostic codes and prescription of ADHD medications. This approach was taken to confirm whether similar outcomes were seen in patients with predominant ADHD and those with predominant other psychiatric disorders, who may not be prescribed ADHD medications. Overall, we observed similar patterns in prevalence and risk ratios regardless of which ADHD definition was used. Both in children and adolescents and in adults, risk ratios for many diseases tended to decrease slightly when ADHD medication was added to the definition of ADHD. In children and adolescents, the reduction in risk ratio was more pronounced for externalizing disorders, SDDs of scholastic skills, and other psychotic disorders. In adults, the reduction in risk ratio was more pronounced for conduct disorders, recurrent depressive disorder, eating disorders, tic disorders, and other neurodevelopmental disorders. This may suggest that patients with ADHD who are prescribed ADHD medication may be at a lower risk of psychiatric comorbidities, or prescription of ADHD medications may have been withheld in patients with specific psychiatric disorders. When ADHD drug prescriptions were included in the definition of ADHD, the risk ratio for ADHD was slightly increased for children and adolescents with many psychiatric disorders. The risk ratio for ADHD remained unchanged or slightly increased for adults with many psychiatric disorders, while the risk ratio for ADHD among adults with some psychiatric disorders, including intellectual disability and tic disorders, decreased. This may indicate that children and adolescents with each psychiatric disorder were at higher risk of being prescribed ADHD medications than the control population, and that the effect was generally relatively smaller in adult patients, because of the higher proportion of adults who were prescribed ADHD medications. It may also be the case that the proportion of ADHD medications prescribed was lower in adults with some psychiatric disorders, such as intellectual disability and tic disorders.

A strength of this study was its design, which included not only a cross-sectional study with 5 analysis years, but also a retrospective cohort study for longitudinal analysis with an observation period of 2 years before and after the initial diagnosis of ADHD. The study had a large population, which included over 45,000 patients with ADHD and over 28,000 patients prescribed ADHD medications in the final study year. The use of the JMDC database enabled tracking of all diagnoses and treatments across various medical hospitals and clinics. In addition, ADHD was defined using two distinct criteria of ICD-10 codes only and ICD-10 code plus prescription of ADHD medications, and the study identified both similarities and differences in results between these definitions. Furthermore, the outcome measures included a large variety of psychiatric comorbidities. This is the first study to use big data to calculate risk ratios for a number of psychiatric disorders in patients with ADHD, to calculate risk ratios for ADHD in patients with psychiatric disorders, and to evaluate the diagnostic sequence of ADHD and psychiatric disorders.

This study also had several limitations. The JMDC database is not generalized and representative in Japan and only includes data from health insurance associations contracted by JMDC. Additionally, the database only includes patients who present to a hospital and therefore may be biased toward patients with more moderate-to-severe symptoms; people with milder symptoms of ADHD may not have been captured in the database. On the other hand, if patients who have belonged to the same health insurance association for a long time are included, there may have been an increased bias toward inclusion of patients with relatively mild symptoms because of the characteristics of the disease. If a patient changed or lost their job and therefore experienced a change in health insurance association, it may not be possible to track their medical history in the JMDC database. The accuracy of data captured for each individual in the database depends on the quality of information entered into the medical records by healthcare professionals. This limitation is common to research using electronic medical records. It is also unclear whether the disease name, once recorded in the medical record, was updated appropriately. It is hoped that the findings obtained in this study will be clarified in prospective studies. Furthermore, it is essential to recognize that the population within the JMDC database, consisting of employed patients and their dependents but not including self-employed people or public servants, may not be directly comparable to other subgroups of the Japanese population. In addition, it has been reported that ADHD in adults is associated with high unemployment in Western countries. In at least one Japanese study, the employment rate of adults with ADHD was numerically lower than that of non-ADHD adults (38). Thus, adults with ADHD may be underrepresented in the JMDC database. Finally, we note that the retrospective cohort study was restricted to 2 years before and after the initial ADHD diagnosis. Increasing the duration of baseline/follow-up periods results in a smaller sample size, so the 2-year baseline and follow-up periods were set to ensure an adequate sample size for the retrospective cohort study.

In conclusion, this population-based study provides a first nationwide assessment of risks of a wide range of psychiatric disorders in patients with ADHD in Japan. ADHD and other psychiatric disorders are comorbid in both children/adolescents and adults in Japan. In particular, ASD in children/adolescents and mood and sleep disorders in adults were commonly diagnosed prior to the initial diagnosis of ADHD.

## Data Availability

The data analyzed in this study were obtained from JMDC Inc. (https://www.jmdc.co.jp/en/) and used under license; therefore, restrictions apply, and the data are not publicly available. Requests to access these datasets should be directed to JMDC, https://www.jmdc.co.jp/en/inquiry/.
