# Supplementary material for: Psychiatric comorbidities of attention deficit/hyperactivity disorder in Japan: a nationwide population-based study
Source: Front Psychiatry. 2024 Oct 24;15:1359872. doi: 10.3389/fpsyt.2024.1359872 (PMC11541049; doi:10.3389/fpsyt.2024.1359872)
Supplement: Supplementary file 1 [file Table1.docx]

Supplementary Material

# Supplementary Methods

**1. ADHD medication drug codes**

| Category | EphMRA-ATC minor  classification code | Ingredient name | Brand name(s) |
| --- | --- | --- | --- |
| MPH | N06B- | Methylphenidate hydrochloride | Concerta® |
| LDX | N06B- | Lisdexamfetamine mesilate | Vyvanse® |
| ATX | N07X | Atomoxetine hydrochloride | Brand name unknown |
|  |  |  | Strattera® |
|  |  |  | Atomoxetine NIPRO |
|  |  |  | Atomoxetine TAKATA |
|  |  |  | Atomoxetine TOWA |
|  |  |  | Atomoxetine DSEP |
|  |  |  | Atomoxetine Sawai |
| GXR | N07X | Guanfacine hydrochloride | Intuniv® |
| GXR | N07X | Guanfacine hydrochloride | Brand name unknown |

EphMRA-ATC, European Pharmaceutical Marketing Research Association-Anatomical Therapeutic Chemical.

**2. Psychiatric disorders ICD-10 codes**

| Psychiatric disorder | ICD-10 classification code(s) |
| --- | --- |
| Substance use disorders | F10–F19 |
| Schizophrenia and schizotypal disorder | F20–F21 |
| Other psychotic disorders | F22–F29 |
| Mood disorders | F30–F39 |
| Bipolar affective disorder | F31 |
| Depressive episode | F32 |
| Recurrent depressive disorder | F33 |
| Anxiety disorders | F40–F41, F93 |
| Obsessive-compulsive disorder | F42 |
| Reaction to severe stress, and adjustment disorders | F43 |
| Dissociative disorders | F44 |
| Somatoform disorders | F45 |
| Eating disorders | F50 |
| Intellectual disability (referred to as ‘mental retardation’ in the ICD-10) | F70–F73, F79 |
| Tic disorders | F95 |
| Sleep disorders | F51, G47 |
| Oppositional defiant disorder | F91.3 |
| Conduct disorders (excluding oppositional  defiant disorder) | F91.0–F91.2, F91.9 |
| Specific developmental disorders of scholastic skills | F81 |
| Specific developmental disorder of motor function | F82 |
| Autism spectrum disorders | F84.0–F84.1, F84.3–F84.5,  F84.8–F84.9 |
| Epilepsy | G40–G41 |

ICD-10, International Statistical Classification of Diseases and Related Health Problems 10th Revision.

**3. Calculations of prevalence and incidence in the overall Japanese population**

The annual prevalence of ADHD from 2017 to 2021 (for ADHD diagnosis only and for diagnosis and medication prescription) and its 95% CI were calculated for the overall analysis population:

$$Annual prevalence of ADHD \left( \% \right)=\frac{Number of disease cases}{Overall population in the year of analysis} \times100$$

In addition to the calculation of the prevalence in the JMDC population, the estimated prevalence adjusted for age (in increments of 1 year) and sex (male, female) distribution in the overall Japanese population was calculated using government statistics data:

$$Estimated prevalence \left( \% \right) in Japanese population aged x to n years= \frac{\left\{ \sum_{i=x}^{n} \left( N_{jmdc male i}^{'}\times\frac{N_{japan male i}}{N_{jmdc male i}} \right)+\sum_{i=x}^{n} \left( N_{jmdc female i}^{'}\times\frac{N_{japan female i}}{N_{jmdc female i}} \right) \right\}}{\sum_{i=x}^{n} N_{japan male i}+\sum_{i=x}^{n} N_{japan female i}}$$

N_japan male/female_: N number in Japan by sex in each analysis year

N_jmdc male/female_: N number in the JMDC database by sex in each analysis year

N’_jmdc male/female_: Number of ADHD patients in the JMDC database by sex in each analysis year

i: Age

When estimating prevalence of ADHD in males only or females only, the number of patients with ADHD of the other sex (ie, females or males, respectively) was excluded from the calculation.

For Population 3, the new incidence rate of ADHD from 2017 to 2021 (for ADHD diagnosis only and for diagnosis and medication prescription) and its 95% CI were calculated:

$$New incidence rate of ADHD \left( \% \right)=\frac{Number of new patients}{Population 3 in the year of analysis} \times100$$

In addition to the calculation of the incidence proportion in the JMDC population, the estimated incidence proportion adjusted for age (in increments of 1 year) and sex (male, female) distribution in the overall Japanese population was calculated using government statistics data:

$$Estimated incidence \left( \% \right)= \frac{\left\{ \sum_{i=x}^{n} \left( N_{jmdc male i}^{'}\times\frac{N_{japan male i}}{N_{jmdc male i}} \right)+\sum_{i=x}^{n} \left( N_{jmdc female i}^{'}\times\frac{N_{japan female i}}{N_{jmdc female i}} \right) \right\}}{\sum_{i=x}^{n} N_{japan male i}+\sum_{i=x}^{n} N_{japan female i}}$$

N_japan male/female_: N number in Japan by sex in each analysis year

N_jmdc male/female_: N number in the JMDC database by sex in each analysis year

N’_jmdc male/female_: Number of new ADHD patients in the JMDC database by sex in each analysis year

i: Age

**Supplementary Table 1** Twelve-month psychiatric comorbidities of ADHD (defined by prescription of ADHD medications in addition to ADHD diagnostic codes) in 2019.

| Psychiatric disorder  Disorder subclassification | Children and adolescents | | | Adults | | |
| --- | --- | --- | --- | --- | --- | --- |
|  | With ADHD (Population 1B)  N = 11,391 | Without ADHD (Population 1B control)^a^  N = 56,955 | Risk ratio  (95% CI) | With ADHD (Population 1B)  N = 9109 | Without ADHD (Population 1B control)^a^  N = 45,545 | Risk ratio  (95% CI) |
| Substance use disorders | 6 (0.1) | 7 (<0.1) | 4.3 (1.4–12.8) | 159 (1.7) | 170 (0.4) | 4.7 (3.8–5.8) |
| Schizophrenia and schizotypal disorder | 1952 (17.1) | 237 (0.4) | 41.2 (36.0–47.1) | 2022 (22.2) | 414 (0.9) | 24.4 (22.0–27.1) |
| Other psychotic disorders | 184 (1.6) | 34 (0.1) | 27.1 (18.8–39.0) | 438 (4.8) | 56 (0.1) | 39.1 (29.6–51.6) |
| Mood disorders | 1324 (11.6) | 306 (0.5) | 21.6 (19.1–24.5) | 5632 (61.8) | 1595 (3.5) | 17.7 (16.8–18.6) |
| Bipolar affective disorder | 354 (3.1) | 49 (0.1) | 36.1 (26.8–48.7) | 1739 (19.1) | 240 (0.5) | 36.2 (31.7–41.4) |
| Depressive episode | 899 (7.9) | 228 (0.4) | 19.7 (17.1–22.8) | 4807 (52.8) | 1400 (3.1) | 17.2 (16.2–18.1) |
| Recurrent depressive disorder | 1 (<0.1) | 0 (0.0) | – | 25 (0.3) | 7 (0.0) | 17.9 (7.7–41.3) |
| Anxiety disorders | 798 (7.0) | 388 (0.7) | 10.3 (9.1–11.6) | 2066 (22.7) | 962 (2.1) | 10.7 (10.0–11.6) |
| OCD | 136 (1.2) | 37 (0.1) | 18.4 (12.8–26.4) | 189 (2.1) | 57 (0.1) | 16.6 (12.3–22.3) |
| Reaction to severe stress, and adjustment disorder | 946 (8.3) | 318 (0.6) | 14.9 (13.1–16.9) | 1019 (11.2) | 291 (0.6) | 17.5 (15.4–19.9) |
| Dissociative disorders | 73 (0.6) | 24 (<0.1) | 15.2 (9.6–24.1) | 375 (4.1) | 77 (0.2) | 24.4 (19.1–31.1) |
| Somatoform disorders | 1059 (9.3) | 626 (1.1) | 8.5 (7.7–9.3) | 695 (7.6) | 492 (1.1) | 7.1 (6.3–7.9) |
| Eating disorders | 40 (0.4) | 54 (0.1) | 3.7 (2.5–5.6) | 71 (0.8) | 54 (0.1) | 6.6 (4.6–9.4) |
| Intellectual disability | 852 (7.5) | 457 (0.8) | 9.3 (8.3–10.4) | 182 (2.0) | 107 (0.2) | 8.5 (6.7–10.8) |
| Tic disorders | 179 (1.6) | 98 (0.2) | 9.1 (7.1–11.7) | 23 (0.3) | 2 (<0.1) | 57.5 (13.6–243.8) |
| Sleep disorders | 1868 (16.4) | 588 (1.0) | 15.9 (14.5–17.4) | 4514 (49.6) | 1997 (4.4) | 11.3 (10.8–11.9) |
| ODD | 99 (0.9) | 8 (<0.1) | 61.9 (30.1–127.1) | 8 (0.1) | 0 (0.0) | – |
| Conduct disorders (excluding ODD) | 60 (0.5) | 8 (<0.1) | 37.5 (17.9–78.4) | 11 (0.1) | 2 (<0.1) | 27.5 (6.1–124.0) |
| SDDs of scholastic skills | 588 (5.2) | 102 (0.2) | 28.8 (23.4–35.5) | 67 (0.7) | 6 (<0.1) | 55.8 (24.2–128.7) |
| SDD of motor function | 197 (1.7) | 148 (0.3) | 6.7 (5.4–8.2) | 9 (0.1) | 8 (<0.1) | 5.6 (2.2–14.6) |
| ASD | 6291 (55.2) | 1333 (2.3) | 23.6 (22.3–24.9) | 1716 (18.8) | 185 (0.4) | 46.4 (39.9–53.9) |
| Epilepsy | 729 (6.4) | 550 (1.0) | 6.6 (5.9–7.4) | 987 (10.8) | 397 (0.9) | 12.4 (11.1–13.9) |

All data are n (prevalence %) unless otherwise stated. ^a^Non-ADHD population, age- and sex-matched to Population 1B (sample size ratio of 5:1). ADHD, attention deficit/hyperactivity disorder; ASD, autism spectrum disorder; OCD, obsessive-compulsive disorder; ODD, oppositional defiant disorder; SDD, specific developmental disorder.

**Supplementary Table 2** ADHD (defined by prescription of ADHD medications in addition to ADHD diagnostic codes) as a 12-month comorbidity of various psychiatric disorders in 2019.

| Psychiatric disorder  Disorder subclassification | Children and adolescents | | | | | Adults | | | | |
| --- | --- | --- | --- | --- | --- | --- | --- | --- | --- | --- |
|  | With each psychiatric disorder  (Population 2) | | Without each psychiatric disorder  (Population 2 control)^a^ | | Risk ratio (95% CI) | With each psychiatric disorder  (Population 2) | | Without each psychiatric disorder  (Population 2 control)^a^ | | Risk ratio (95% CI) |
|  | Pop | ADHD prevalence, n (%) | Pop | ADHD prevalence, n (%) |  | Pop | ADHD prevalence, n (%) | Pop | ADHD prevalence, n (%) |  |
| Substance use disorders | 271 | 6 (2.2) | 542 | 1 (0.2) | 12.0 (1.5–99.2) | 15,510 | 159 (1.0) | 31,020 | 57 (0.2) | 5.6 (4.1–7.5) |
| Schizophrenia and schizotypal disorder | 6395 | 1952 (30.5) | 12,790 | 118 (0.9) | 33.1 (27.5–39.7) | 42,911 | 2022 (4.7) | 85,822 | 130 (0.2) | 31.1 (26.1–37.1) |
| Other psychotic disorders | 745 | 184 (24.7) | 1490 | 13 (0.9) | 28.3 (16.2–49.3) | 6504 | 438 (6.7) | 13,008 | 27 (0.2) | 32.4 (22.0–47.8) |
| Mood disorders | 8173 | 1324 (16.2) | 16,346 | 109 (0.7) | 24.3 (20.0–29.5) | 178,468 | 5632 (3.2) | 356,936 | 225 (0.1) | 50.1 (43.8–57.2) |
| Bipolar affective disorder | 1348 | 354 (26.3) | 2696 | 19 (0.7) | 37.3 (23.6–58.8) | 26,636 | 1739 (6.5) | 53,272 | 71 (0.1) | 49.0 (38.7–62.1) |
| Depressive episode | 6421 | 899 (14.0) | 12,842 | 77 (0.6) | 23.4 (18.5–29.4) | 158,694 | 4807 (3.0) | 317,388 | 259 (0.1) | 37.1 (32.8–42.1) |
| Recurrent depressive disorder | 6 | 1 (16.7) | 12 | 0 (0.0) | – | 843 | 25 (3.0) | 1686 | 1 (0.1) | 50.0 (6.8–368.4) |
| Anxiety disorders | 9399 | 798 (8.5) | 18,798 | 131 (0.7) | 12.2 (10.1–14.6) | 113,233 | 2066 (1.8) | 226,466 | 293 (0.1) | 14.1  (12.5–15.9) |
| OCD | 944 | 136 (14.4) | 1888 | 11 (0.6) | 24.7 (13.4–45.5) | 5198 | 189 (3.6) | 10,396 | 19 (0.2) | 19.9 (12.4–31.8) |
| Reaction to severe stress, and adjustment disorders | 7268 | 946 (13.0) | 14,536 | 119 (0.8) | 15.9 (13.2–19.2) | 25,096 | 1019 (4.1) | 50,192 | 95 (0.2) | 21.5 (17.4–26.5) |
| Dissociative disorders | 1064 | 73 (6.9) | 2128 | 10 (0.5) | 14.6 (7.6–28.2) | 9475 | 375 (4.0) | 18,950 | 32 (0.2) | 23.4 (16.3–33.6) |
| Somatoform disorders | 15,545 | 1059 (6.8) | 31,090 | 235 (0.8) | 9.0 (7.8–10.4) | 56,366 | 695 (1.2) | 112,732 | 160 (0.1) | 8.7 (7.3–10.3) |
| Eating disorders | 2006 | 40 (2.0) | 4012 | 15 (0.4) | 5.3 (3.0–9.6) | 3860 | 71 (1.8) | 7720 | 21 (0.3) | 6.8 (4.2–11.0) |
| Intellectual disability | 11,458 | 852 (7.4) | 22,916 | 162 (0.7) | 10.5  (8.9–12.4) | 5880 | 182 (3.1) | 11,760 | 40 (0.3) | 9.1 (6.5–12.8) |
| Tic disorders | 2028 | 179 (8.8) | 4056 | 38 (0.9) | 9.4 (6.7–13.3) | 286 | 23 (8.0) | 572 | 1 (0.2) | 46.0 (6.2–338.9) |
| Sleep disorders | 16,802 | 1868 (11.1) | 33,604 | 216 (0.6) | 17.3 (15.0–19.9) | 257,711 | 4514 (1.8) | 515,422 | 369 (0.1) | 24.5 (22.0–27.2) |
| ODD | 202 | 99 (49.0) | 404 | 4 (1.0) | 49.5 (18.5–132.6) | 13 | 8 (61.5) | 26 | 0 (0.0) | – |
| Conduct disorders (excluding ODD) | 205 | 60 (29.3) | 410 | 3 (0.7) | 40.0 (12.7–126.0) | 83 | 11 (13.3) | 166 | 0 (0.0) | – |
| SDDs of scholastic skills | 2093 | 588 (28.1) | 4186 | 45 (1.1) | 26.1 (19.4–35.2) | 350 | 67 (19.1) | 700 | 1 (0.1) | 134.0 (18.7–961.3) |
| SDD of motor function | 6955 | 197 (2.8) | 13,910 | 50 (0.4) | 7.9 (5.8–10.7) | 325 | 9 (2.8) | 650 | 0 (0.0) | – |
| ASD | 35,756 | 6291 (17.6) | 71,512 | 331 (0.5) | 38.0 (34.1–42.4) | 10,670 | 1716 (16.1) | 21,340 | 55 (0.3) | 62.4 (47.8–81.5) |
| Epilepsy | 13,543 | 729 (5.4) | 27,086 | 205 (0.8) | 7.1 (6.1–8.3) | 38,769 | 987 (2.5) | 77,538 | 138 (0.2) | 14.8  (12.0–17.1) |

^a^Age- and sex-matched to Population 2 (sample size ratio of 2:1). ADHD, attention deficit/hyperactivity disorder; ASD, autism spectrum disorder; OCD, obsessive-compulsive disorder; ODD, oppositional defiant disorder; Pop, population; SDD, specific developmental disorder.

**Supplementary Figure 1** Annual prevalence **(A, B)** and incidence **(C, D)** of ADHD defined by prescription of ADHD medications in addition to ADHD diagnostic codes. Circles and solid line: males; squares and dotted line: females.

ADHD, attention deficit/hyperactivity disorder.


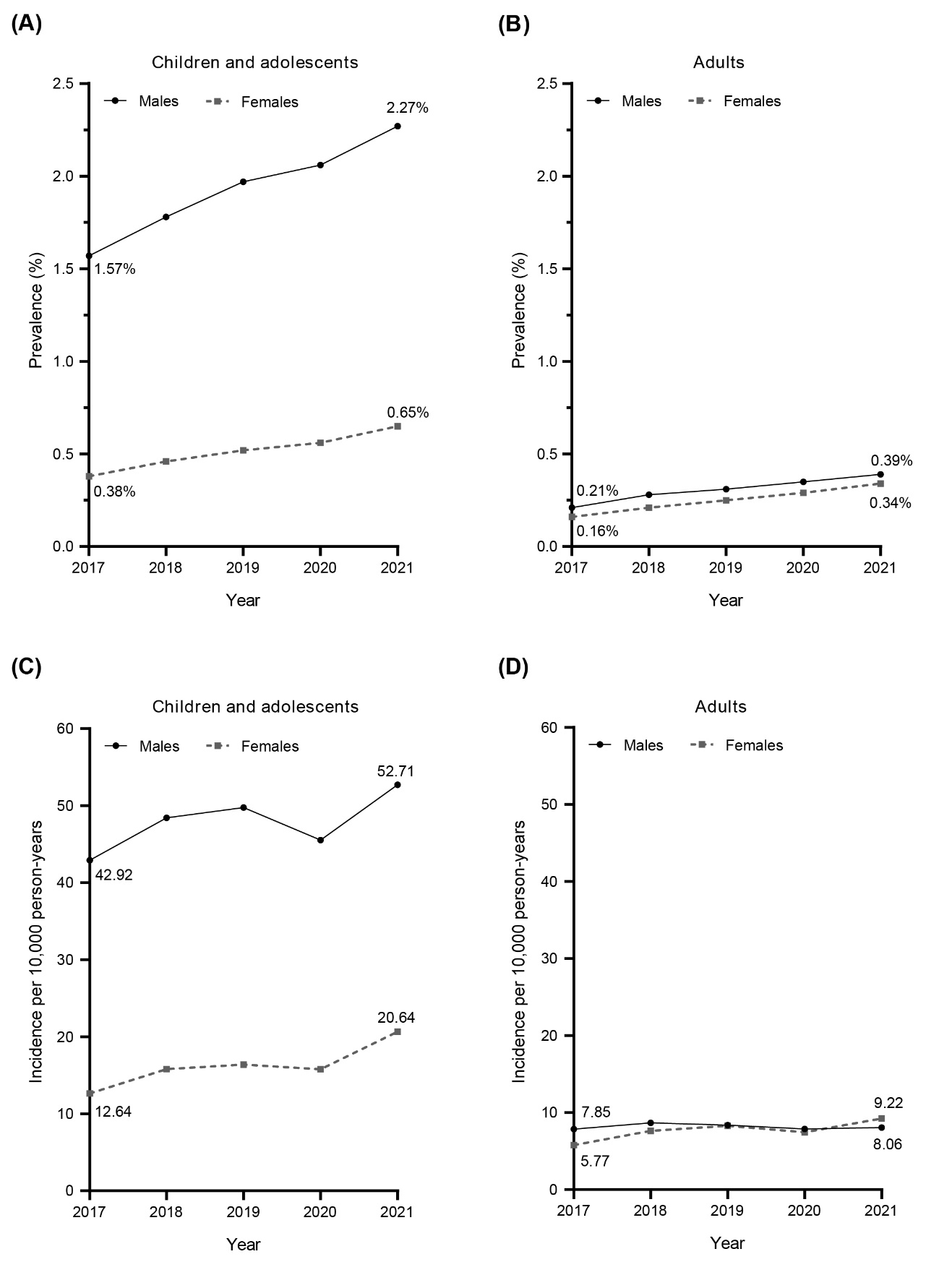


**Supplementary Figure 2** Psychiatric disorders diagnosed before (black columns) and after (grey columns) initial prescription of ADHD medication (plus ADHD diagnosis). Tables show summary statistics for the number of psychiatric disorders diagnosed before and after the initial prescription of ADHD medications.

ADHD, attention deficit/hyperactivity disorder; ASD, autism spectrum disorder; max, maximum; min, minimum; OCD, obsessive-compulsive disorder; ODD, oppositional defiant disorder; SDD, specific developmental disorder; Q, Quartile.


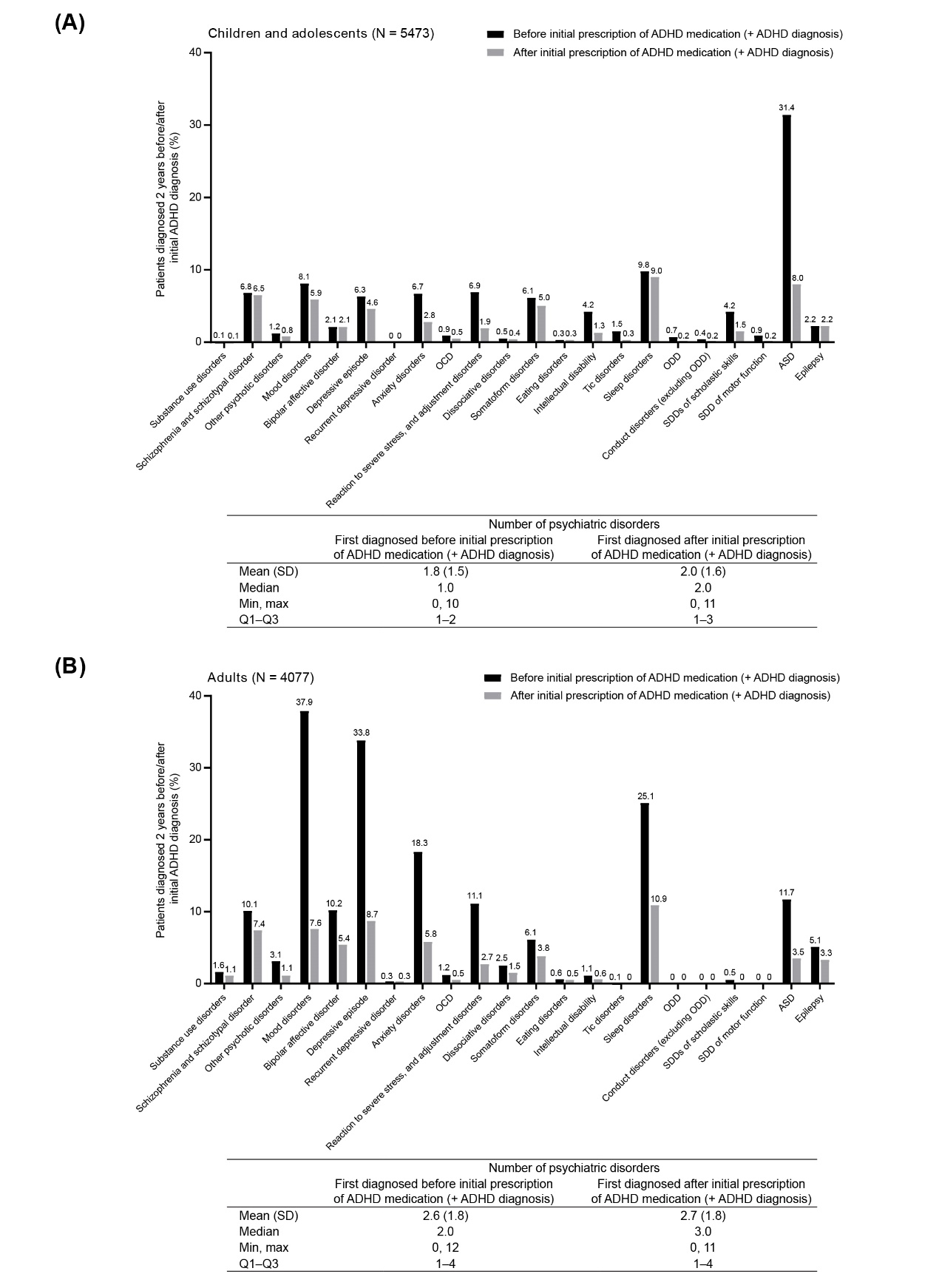


**Supplementary Figure 3** Intervals between diagnoses in people with a diagnosis of ADHD and diagnoses of psychiatric disorders. In children and adolescents, intervals between **(A)** psychiatric disorder diagnoses and the initial ADHD diagnosis and **(B)** the initial ADHD diagnosis and diagnoses of other psychiatric disorders are shown. In adults, intervals between **(C)** psychiatric disorder diagnoses and the initial ADHD diagnosis and **(D)** the initial ADHD diagnosis and diagnoses of other psychiatric disorders are shown. Red lines indicate median values; black crosses indicate mean values; grey rectangles show the range from Q1 to Q3; error bars show maximum and minimum values.

ADHD, attention deficit/hyperactivity disorder; Q, quartile.


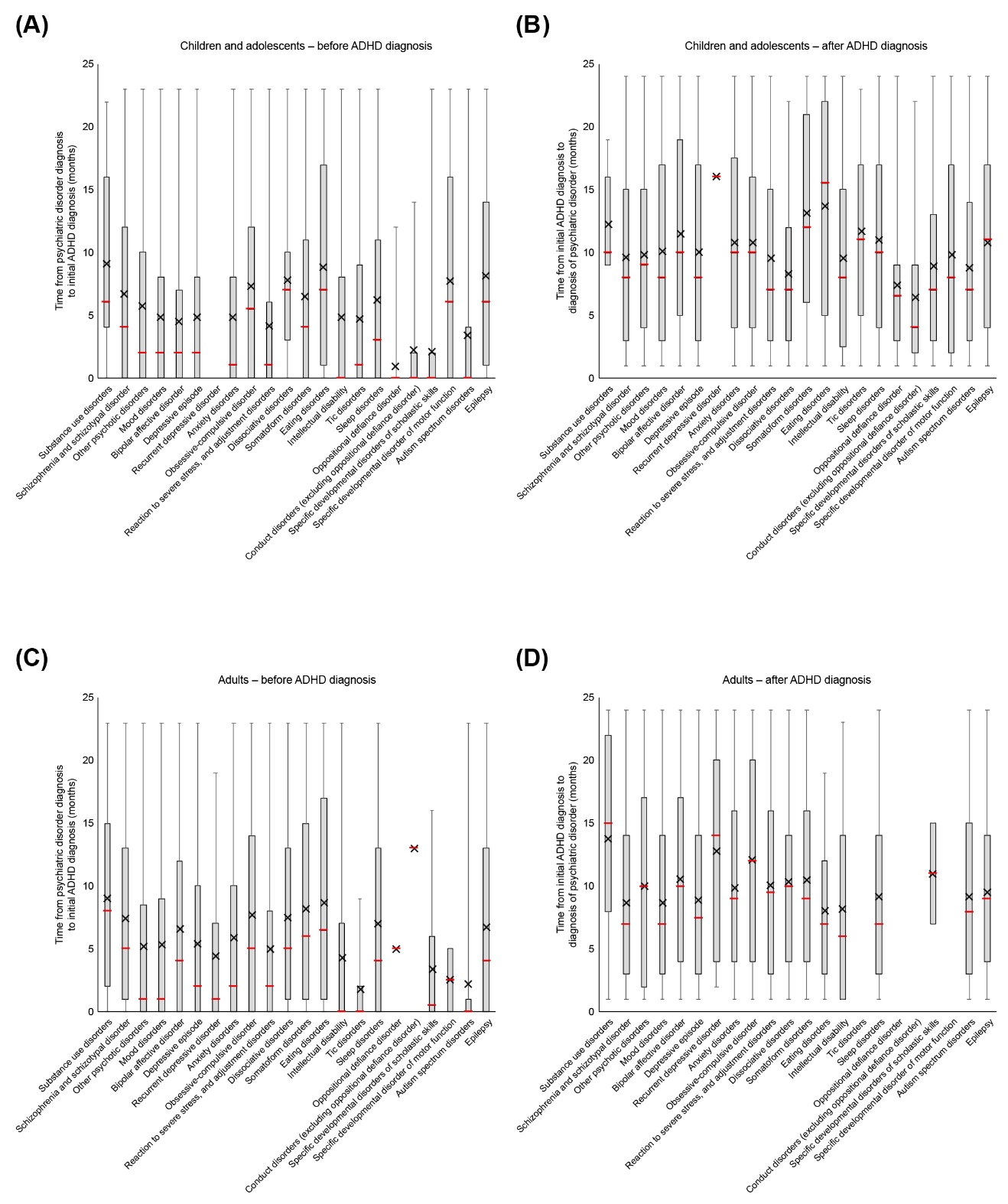


**Supplementary Figure 4** Intervals between diagnoses in people with a diagnosis of ADHD and prescribed ADHD medications and with diagnoses of psychiatric disorders. In children and adolescents, intervals between **(A)** psychiatric disorder diagnoses and the initial prescription of ADHD medication with ADHD diagnosis and **(B)** the initial prescription of ADHD medication with ADHD diagnosis and diagnoses of other psychiatric disorders are shown. In adults, intervals between **(C)** psychiatric disorder diagnoses and the initial prescription of ADHD medication with ADHD diagnosis and **(D)** the initial prescription of ADHD medication with ADHD diagnosis and diagnoses of other psychiatric disorders are shown. Red lines indicate median values; black crosses indicate mean values; grey rectangles show the range from Q1 to Q3; error bars show maximum and minimum values.

ADHD, attention deficit/hyperactivity disorder; Q, Quartile.


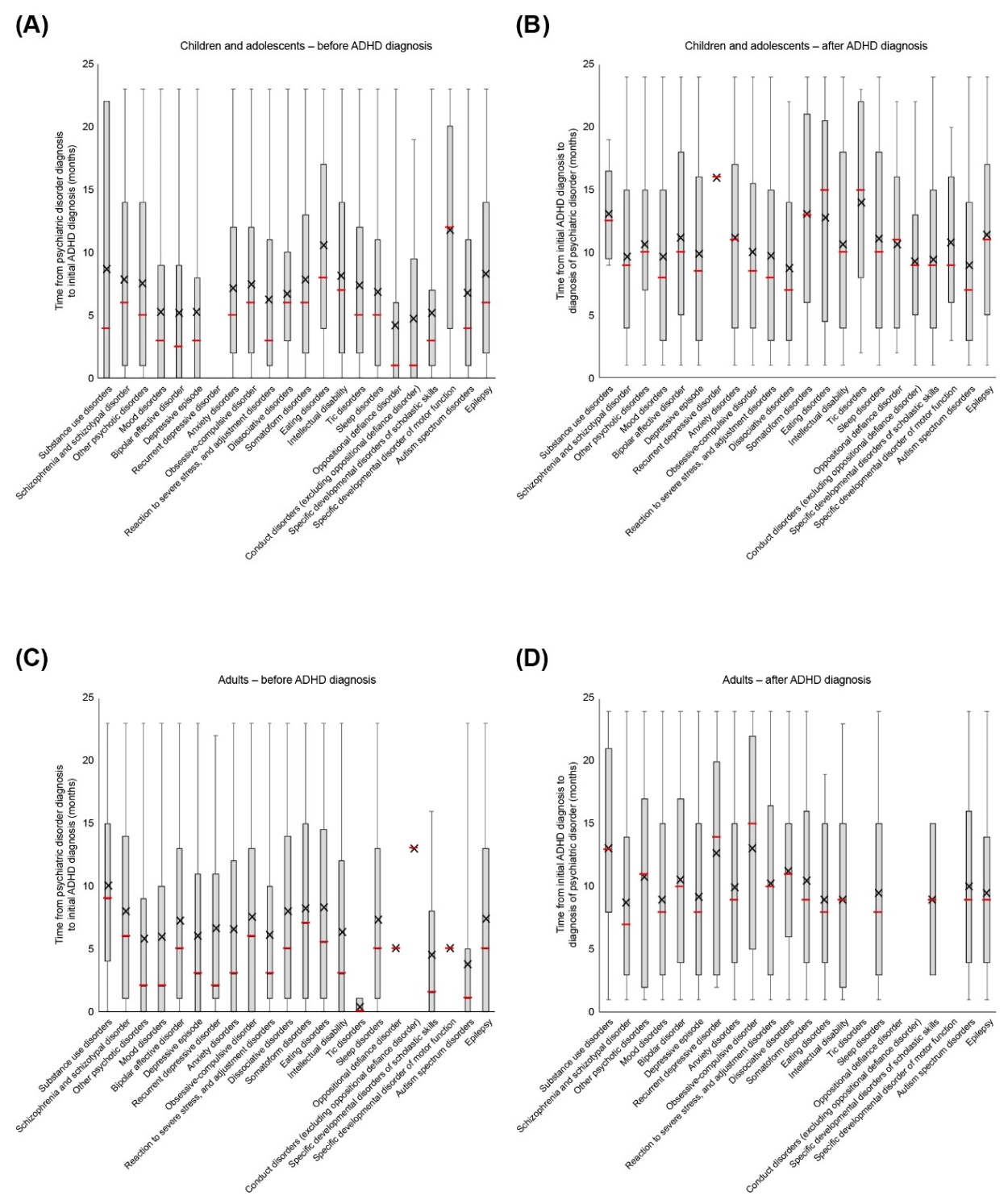


**
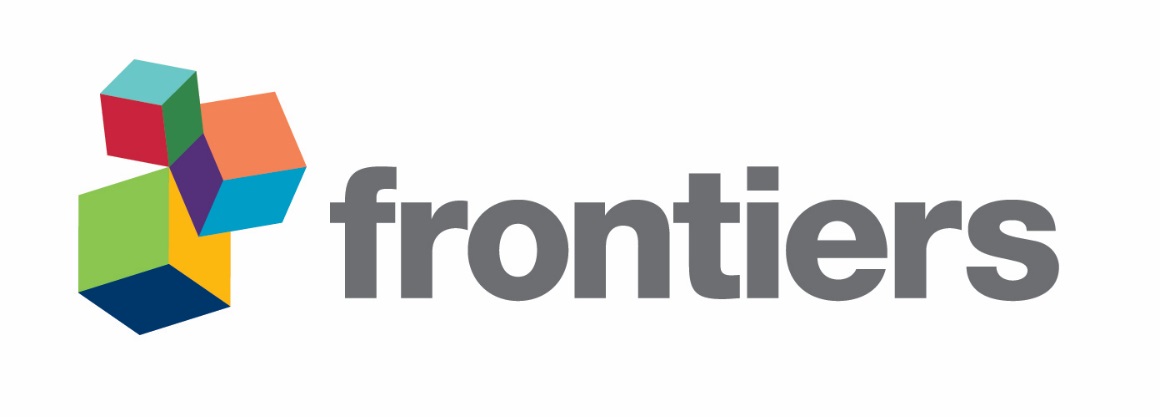
**
